# Multidimensional single-cell analysis reveals immune dysfunction and inflammatory response in lymphatic malformations

**DOI:** 10.1093/procel/pwaf103

**Published:** 2025-11-22

**Authors:** Chunxiao Chen, Wenhao Ju, Xueying Li, Kexin Yao, Jun Cao, Songqi Duan, Xueqi Lv, Tianli Zhang, Sanlin Li, Jiawen Li, Feng He, Baofa Sun, Gang Shen

**Affiliations:** Vascular Anomalies and Vascular Interventional Center, Capital Center for Children’s Health, Capital Medical University, Capital Institute of Pediatrics, Beijing 100020, China; State Key Laboratory of Medicinal Chemical Biology, Frontiers Science Center for Cell Responses, College of Life Sciences, Nankai University, Tianjin 300071, China; State Key Laboratory of Cardiovascular Disease, Department of Structural Heart Disease, National Center for Cardiovascular Disease, Fuwai Hospital, National Center for Cardiovascular Diseases, Key Laboratory of Innovative Cardiovascular Devices, Chinese Academy of Medical Sciences, Chinese Academy of Medical Sciences and Peking Union Medical College, Beijing 100037, China; State Key Laboratory of Medicinal Chemical Biology, Frontiers Science Center for Cell Responses, College of Life Sciences, Nankai University, Tianjin 300071, China; State Key Laboratory of Medicinal Chemical Biology, Frontiers Science Center for Cell Responses, College of Life Sciences, Nankai University, Tianjin 300071, China; College of Chemistry and Life Science, Beijing University of Technology, Beijing 100124, China; College of Food Science, Sichuan Agricultural University, Ya’an 625014, China; State Key Laboratory of Medicinal Chemical Biology, Frontiers Science Center for Cell Responses, College of Life Sciences, Nankai University, Tianjin 300071, China; State Key Laboratory of Medicinal Chemical Biology, Frontiers Science Center for Cell Responses, College of Life Sciences, Nankai University, Tianjin 300071, China; Vascular Anomalies and Vascular Interventional Center, Capital Center for Children’s Health, Capital Medical University, Capital Institute of Pediatrics, Beijing 100020, China; Vascular Anomalies and Vascular Interventional Center, Capital Center for Children’s Health, Capital Medical University, Capital Institute of Pediatrics, Beijing 100020, China; Department of Biochemistry and Immunology, Capital Center for Children’s Health, Capital Medical University, Capital Institute of Pediatrics, Beijing 100020, China; State Key Laboratory of Medicinal Chemical Biology, Frontiers Science Center for Cell Responses, College of Life Sciences, Nankai University, Tianjin 300071, China; Vascular Anomalies and Vascular Interventional Center, Capital Center for Children’s Health, Capital Medical University, Capital Institute of Pediatrics, Beijing 100020, China

**Keywords:** lymphatic malformations, single-cell RNA sequencing, immune dysfunction, inflammatory response

## Abstract

Lymphatic malformations (LMs) are debilitating and potentially life-threatening diseases. However, the immune phenotype of circulating cells and underlying molecular mechanisms in LMs remain poorly understood. Here, we performed integrated single-cell RNA, T-cell receptor, and B-cell receptor sequencing (scRNA-seq, scTCR-seq, and scBCR-seq) of peripheral blood and pleural effusion from patients with LMs to delineate their immune landscape. We identified an expansion of pro-inflammatory CD14^+^CD16^+^ monocytes and atypical memory B cells, accompanied by reduced cytotoxic CD8^+^ T and natural killer (NK) cells. Functional analysis revealed impaired antigen processing and presentation in CD14^+^ monocytes, and dysregulated transcription factor activity, potentially driving immune dysfunction. Additionally, LMs exhibited substantial remodeling of TCR and BCR repertoires, with shifts in clonality and diversity. Moreover, the CXCL16–CXCR6 interaction was associated with inflammatory responses, while upregulation of the inhibitory checkpoint HLA-E: CD94-NKG2A potentially contributed to impaired NK cell activity. Finally, we constructed a shared pro-inflammatory monocyte program and revealed S100A8 as a potential therapeutic target for LMs. We further demonstrated that S100A8 pharmacological inhibition could ameliorate the pathological phenotype of LMs. Collectively, our findings delineate cell type-specific immune dysregulation in LMs, offering insights for therapeutic development.

## Introduction

**Figure 1.**
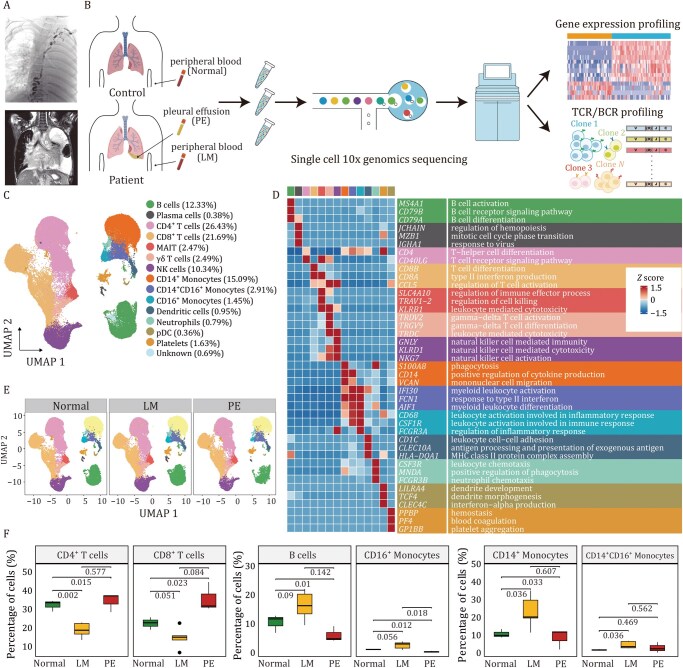
**The transcriptional landscape of immune cells in peripheral blood and pleural effusion samples**. (A) Representative lymphangiography (upper) and magnetic resonance imaging (MRI, bottom) images of a patient with central conducting lymphatic anomaly (CCLA), showing prominently dilated and tortuous central lymphatic channels. (B) Schematic overview of the study design, including sample sources, cell sorting strategy, and workflow. Schematics were created with BioRender, with some elements from 10x Genomics (C) UMAP visualization of major immune cell populations in peripheral blood and pleural effusion; values in parentheses indicate the proportion of each cell type. (D) Heatmap showing relative expression levels of canonical marker genes and representative functional signatures (derived from GO enrichment of hypervariable genes). (E) UMAP plots illustrating the distribution of immune cell types in the Normal (blood from healthy donors), LM (blood from patients with LMs), and PE (pleural effusion from patients with LMs) groups. (F) Box plots showing the percentage of each cell type across the three groups. Data are presented as mean ± SD. Statistical significance was determined using a two-tailed unpaired Student’s *t*-test; normal: *n *= 3, LM: *n *= 5, PE: *n *= 3.

Lymphatic vessels maintain tissue fluid homeostasis by draining interstitial fluid into the bloodstream and serve as conduits for immune cell trafficking, which is essential for immune surveillance (Oliver et al., 2020; Petrova and Koh, 2020). Malformations in the construction of the lymphatic vasculature during embryonic development can cause severe systemic consequences, including fetal mortality due to the adverse effects of excessive fluid accumulation on the development and functioning of the pulmonary and cardiac systems (Trenor and Chaudry, 2014). Lymphatic malformations (LMs) are thought to arise from the abnormal development of the lymphatic system, typically presenting at birth or early infancy as fluid-filled soft tissue masses (Makinen et al., 2021). Our research focuses on central conducting lymphatic anomaly (CCLA) (Fig. 1A) which is characterized by thoracic duct dysfunction and retrograde lymphatic flow (Aspelund et al., 2016; Fang et al., 2000). CCLA represents a severe form of LMs with limited effective treatments available (Itkin et al., 2020; Trenor and Chaudry, 2014). Recent studies have implicated deficient *EPHB4* function caused by mutations in the pathological dilatation of central lymphatic vessels (Wang et al., 2010b), which disrupts normal lymphatic drainage and causes reflux. Additionally, gain-of-function variants in *ARAF* and other RAS/MAPK pathway genes have been implicated in driving CCLA pathogenesis via MAPK hyperactivation (Li et al., 2019a). These malformations can lead to conditions such as chylothorax, pleural effusion (PE), or pericardial effusion, lymphangiectasia, and lymphedema (Brouillard et al., 2014; Li et al., 2022; Liu et al., 2023; Petkova et al., 2023; Winkler et al., 2022).

The initiation and progression of LMs may involve multiple cell types, while previous studies have primarily focused on lymphatic endothelial cells (LECs) (Petkova et al., 2023). In recent years, emerging evidence underscores immune-vascular interactions in vascular pathologies. For instance, single-cell landscape of peripheral blood from Kawasaki disease (Wang et al., 2021) or pulmonary hypertension (Wu et al., 2023) has revealed distinctive characteristics of innate and adaptive immune responses, offering mechanistic and therapeutic insights into vascular disease. Moreover, patients with LMs typically exhibit features such as coagulation abnormalities (Oliver et al., 2020), nutritional deficiencies (Makinen et al., 2021), and compromised immune system (Betterman and Harvey, 2016), which could lead to weakened immune function. Notably, compared to normal tissue, LMs exhibit higher levels of interferon expression at both mRNA and protein levels, and enhanced activation of interferon signaling, thereby inducing chronic inflammation (Fleming et al., 2008). However, the influence of LMs on the reprogramming of the immune system has not been thoroughly defined. Therefore, exploring the composition and functional status of immune cells from the peripheral blood and PE in LMs is essential. Advances in single-cell RNA sequencing (scRNA-seq) enable unbiased profiling of immune dysregulation at cellular resolution (Pai and Satpathy, 2021; Stubbington et al., 2017), providing an unprecedented opportunity to define LMs-specific immunopathology.

In this work, we performed scRNA-seq, T-cell receptor sequencing, and B-cell receptor sequencing (scTCR-seq and scBCR-seq) to generate a comprehensive transcriptional landscape of immune cells and to decipher their cellular characteristics in LMs. We identified 14 major cell types and analyzed differentially expressed genes (DEGs), which revealed immune cell dysfunction and increased inflammatory response in LMs. We further discovered that the activity of the transcription factors (TFs) REL and RFX5 in CD14^+^ monocytes was decreased in patients, which was associated with a downregulated capacity for antigen processing and presentation. Cytotoxic effector populations (GZMB^+^CD8^+^ T cells and CD56^high^CD16^low^ natural killer [NK] cells) showed diminished proportions and functional impairment, with trajectory analysis indicating a differentiation blockade. Intercellular communication analysis identified specific CXCL16–CXCR6 interactions between monocytes and mucosal-associated invariant T (MAIT) cells, which have been reported to participate in inflammatory responses. Strikingly, we identified a Shared CD14^+^CD16^+^ Monocyte Expression Program (SMEP) both in the LM and PE groups, indicating a convergent pathological mechanism. Our analysis revealed that several Food and Drug Administration (FDA)-approved drugs could target key genes within SMEP, such as *S100A8*, *FCGR1A*, and *CD163*. Furthermore, genetic ablation of *Vegfr3* in mice recapitulated the lymphangiogenic phenotype, which was significantly attenuated by treatment with paquinimod, an S100A8 inhibitor. These results suggested that targeting *S100A8* may represent a promising therapeutic strategy. Together, our findings help elucidate disease mechanisms by revealing transcriptomic profiles, cell differentiation states, and cellular interactions, thereby identifying cell type-based markers and pathways for immune dysregulation and potential therapeutic targets.

## Results

### High-resolution mapping of the immune cell landscape in LMs using scRNA-seq

To obtain an unbiased and comprehensive understanding of the immunological features associated with disease status in LMs, we performed scRNA-seq to map a high-­resolution immune cellular landscape. We collected five peripheral blood samples (labeled as LM), and three PE samples (labeled as PE) from patients with LMs, as well as three peripheral blood samples from healthy donors (labeled as Normal) (Fig. 1B; Table S1 and Methods). Whole-exome sequencing (WES) revealed that two of three patients carried somatic mutations in *EPHB4* (Fig. S1A; Table S2 and Methods). Both mutations were protein-truncating variants located in exon 17 of the *EPHB4* gene (RefSeq: NM_004444) and were highly likely to result in loss of function. This observation is consistent with a previous study reporting *EPHB4* mutations as a causal factor of CCLA (Li et al., 2018).

For the scRNA-seq data, after quality control and filtering, a total of 92,088 cells were obtained for subsequent analysis, comprising 73,770 cells from peripheral blood (Normal: 33,264 cells; LM: 40,506 cells) and 18,318 cells from PE. We integrated the filtered data using the fast mutual nearest neighbors (MNNs) method to correct for batch effects and then applied an unsupervised graph-based clustering algorithm to identify distinct populations. Initial clustering identified major cell populations based on canonical marker expression and functional annotations (Figs. 1C, 1D and S1B–D; Table S3), including lymphocytes (CD4^+^ T cells, CD8^+^ T cells, MAIT cells, gamma delta [γδ] T cells, NK cells, B cells, and plasma cells), as well as myeloid cells (CD14^+^ monocytes, CD14^+^CD16^+^ monocytes, CD16^+^ monocytes, neutrophils, dendritic cells, plasmacytoid dendritic cells [pDCs] and platelets). All identified cell types originated from multiple samples, suggesting that the cells were grouped based on immune-associated characteristics rather than patient specificity (Figs. 1E and S1E). Proportional analysis revealed disease-associated shifts: both CD4^+^ and CD8^+^ T cells were significantly reduced in the LM group compared to the Normal group, while B cells and monocytes were increased in the LM group (Fig. 1F). Interestingly, among the immune cell types, T cells were significantly enriched in PE, while NK cells were predominantly enriched in peripheral blood (Fig. S1F and S1G). These results reveal the immune cell composition and the dysregulation of cell proportions in LMs.

### Cell type-specific transcriptional alterations underlying immune dysfunction in LMs

**Figure 2.**
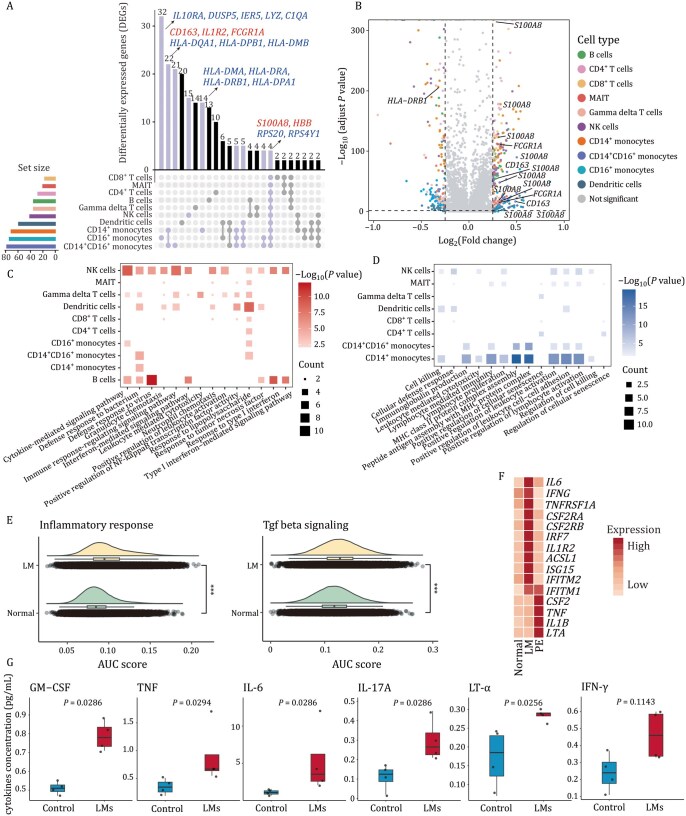
**Transcriptional alterations in immune functions across major cell types between the Normal and LM groups**. (A) UpSet plot showing the intersections of DEGs among major immune cell types between the Normal and LM groups. Dots indicate shared genes among cell types. The upper bars show intersection sizes, the left bars show total gene counts per cell type, and representative key genes are highlighted above. (B) Volcano plot displaying DEGs across cell types. Each point represents an individual gene, colored by its associated cell type. Gray points denote genes without significant expression differences between the Normal and LM groups. (C and D) Heatmap showing significantly enriched GO terms representing disease-related pathways that were upregulated (C) or downregulated (D) in the LM group compared with the Normal group. (E) Ridge plots showing the AUC score of the pathways in the two groups. Two-sided Wilcoxon rank-sum tests. (F) Heatmap showing relative expression levels of pro-inflammatory cytokines across the three groups. (G) Box plots comparing serum cytokine levels from donors with LMs or healthy controls. Each dot represents one biological replicate (*n *= 4 per group), two-sided Wilcoxon rank-sum tests. ***P < 0.001.

To delineate shared and cell type-specific transcriptional alterations in LMs, we conducted an analysis of DEGs across the major immune cell types at two levels (LM vs. normal, PE vs. LM; Table S4 and Methods). By comparing the number of DEGs, we identified that the difference between PE and LM groups was greater than that between two blood groups (LM and Normal) (Figs. 2A and S2A). Additionally, three monocyte subtypes (CD14^+^ monocytes, CD14^+^CD16^+^ monocytes, and CD16^+^ monocytes) exhibited pronounced transcriptional alterations, as evidenced by their higher DEG numbers. Interestingly, we identified four genes with consistent expression changes across all cell types, including a key mediator *S100A8* (Wang et al., 2018) of intracellular inflammatory signaling (Fig. 2B). Furthermore, upregulated DEGs exhibited significant enrichment in immune regulation, cytokine signaling, and interferon signaling (Fig. 2C), implying immune dysregulation and chronic inflammation. Conversely, the downregulated DEGs were related to antigen presentation, leukocyte activation, and NK cell-mediated cytotoxicity (Fig. 2D), indicating a potential deficit in the immune response to external stimuli. In contrast to Normal group, cells from LM group exhibited a globally enhanced inflammatory signature, characterized by marked upregulation of pro-inflammatory cytokines (Fig. 2E and 2F). We further confirmed the coexisting hyperinflammation in patients, suggesting that sustained inflammation is a significant pathological feature of LMs (Fig. 2G).

On the other hand, PE group showed unique enrichment in hypoxia, DNA damage, and inflammation, while the chemotactic and migratory abilities of immune cells were significantly decreased (Fig. S2A–D). Especially, *DDIT4* (a hypoxia-inducible gene) was upregulated among all lymphocyte cell types (Fig. S2E), which was involved in pathways related to hypoxia, DNA damage, gene transcription, and PI3K-Akt signaling pathway. In contrast, *GNAI2* (which participates in the regulation of chemotaxis and cell migration) was downregulated in all immune cell types. It has been reported that B cells from Gnai2^−/−^ mice enter lymph nodes poorly and move more slowly than do wild-type B cells (Han et al., 2005) (Fig. S2F). Moreover, gene set enrichment analysis (GSEA) reinforced PE-specific chemotaxis and migration defects (Fig. S2G and Methods), suggesting immune cell trapping within effusion microenvironments. Taken together, these results unveil a heightened inflammatory response and immune dysfunction in peripheral blood, along with impaired migration of immune cells from lymph nodes in their PE.

### Enhanced inflammation and impaired antigen presentation in myeloid cells

**Figure 3.**
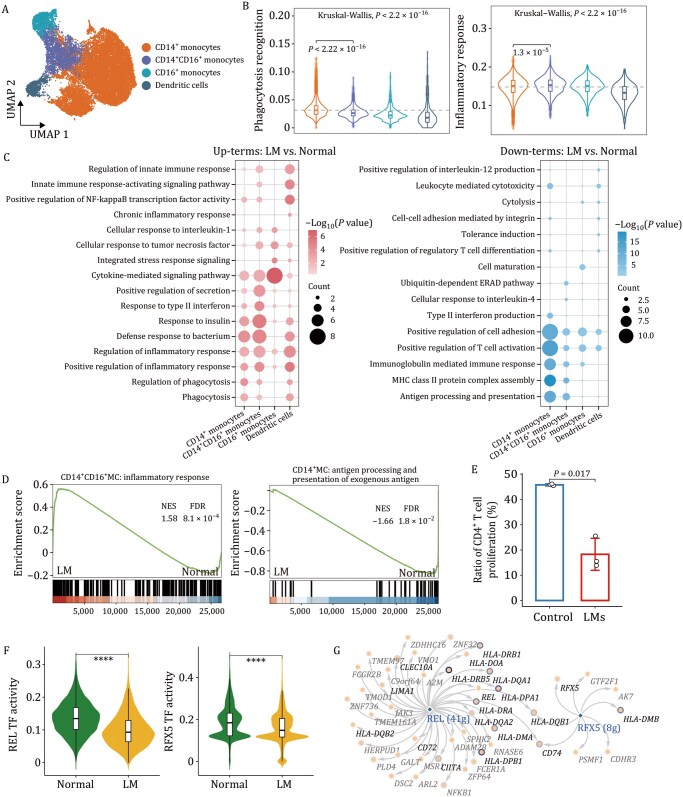
**LM-related transcriptional alterations in various subtypes of myeloid cells**. (A) UMAP plot depicting myeloid cells annotated by subtype. (B) Violin plots showing the pathway scores across myeloid subtypes. Statistical significance was assessed using Kruskal–Wallis tests for overall group differences, with post-hoc Wilcoxon rank-sum tests for pairwise comparisons between designated subtypes. Dashed lines indicate mean scores across all groups. (C) Bubble plots showing enriched pathways for each myeloid subtype identified by GO analysis of upregulated (left) and downregulated (right) DEGs, with bubble size indicating gene counts and color representing statistical significance. (D) GSEA plots of representative pathways in myeloid subtypes, comparing the Normal and LM groups. (E) Ratio of proliferating CD4^+^ T cells after co-culture with monocytes from donors with LMs or healthy controls. Each dot represents one biological replicate (*n *= 3 per group). Bars indicate mean ± SD. Statistical comparison was performed with a two-tailed unpaired *t*-test. (F) Violin plots showing the activities of the TFs REL and RFX5 comparing the LM and the Normal groups. Comparisons were performed using two-tailed unpaired Student’s *t*-test. (G) Transcriptional regulatory network of differentially expressed transcription factors (TFs) REL and RFX5 (colored in blue) and their target genes in CD14^+^ monocytes. Downregulated DEGs which involved in antigen processing and presentation are colored in black. The intensity of the blue border around each circle corresponds to the degree of downregulation in the LM group. ****P < 0.0001.

To investigate the impact of LMs on different myeloid subtypes, unsupervised clustering analysis identified four clusters with distinct gene signatures (Figs. 3A, S3A and S3B). Cells within each subtype based on the transcriptional coherence, forming a pseudo-temporal trajectory that progressed sequentially from CD14^+^ monocytes (classical monocytes) through CD14^+^CD16^+^ monocytes (intermediate monocytes), culminating in two terminal clusters: CD16^+^ monocytes (non-classical monocytes) and dendritic cells (Fig. S3C and Methods). As expected, CD14^+^ monocytes were predominantly among the myeloid cells, whereas CD16^+^ monocytes showed significant depletion in the PE group (Figs. 1F and S3D). Notably, CD14^+^ monocytes exhibited high activity of phagocytosis-like recognition, while CD14^+^CD16^+^ monocytes were related to inflammatory response (Figs. 3B and S3E). Compared to the Normal group, we revealed enhanced inflammatory response of CD14^+^ monocytes and CD14^+^CD16^+^ monocytes in the LM group, especially in CD14^+^CD16^+^ monocytes (Fig. 3C and 3D). Conversely, the capacity for antigen processing and presentation in myeloid cells was significantly reduced, especially in CD14^+^ monocytes (Figs. 3C, 3D, S3F and Methods), implying a diminished immune response to external antigen stimulation. We further verified the reduced antigen presentation ability of monocytes in LMs by *in vitro* functional assays (Figs. 3E, S3G and Methods).

To identify master regulators governing antigen processing and presentation capabilities in CD14^+^ monocytes, we constructed gene regulatory networks integrating TFs with their target genes (Methods). This analysis revealed that the activity of several TFs was decreased in the LM group (Fig. S3H). We further focused on two TFs (REL and RFX5), whose activity were significantly downregulated in the LM group, and regulated numerous target genes that were involved in antigen processing and presentation (Fig. 3F and 3G). Consistently, the activity of REL and RFX5 was positively correlated with the expression of target genes (Fig. S3I), suggesting these two TFs may serve as dominant factors in regulating antigen processing and presentation in LMs. Overall, our findings reveal molecular characteristics associated with LMs in myeloid cells, particularly highlighting two key features in CD14^+^ monocytes: enhanced generalized inflammatory responses coupled with diminished antigen processing and presentation capacity, making them significant and potentially defining hallmarks.

### Dysfunctional atypical memory B cells undergo abnormal expansion in LMs

A hallmark of the adaptive immune system is its capacity to generate memory B cells (MBCs) that confer protective immunity against recurrent pathogens (Wienands and Engels, 2016). Classical memory B cells (cMBCs) were annotated as plasma cells due to high expression of *CD27*, *JCHAIN*, and *MZB1* (Fig. S4A and S4B). Referred to as atypical MBCs (atMBCs), or called exhausted B cells, these can be distinguished from cMBCs by lacking *CD27* expression and exhibiting upregulated inhibitory receptors, including *FCRL5*, *ITGAX*, and *FCRL3* (Fig. S4B and S4C). In our data, atMBCs highly expressed immunoglobulin G (IgG) molecules, consistent with their role in adaptive immunity and IgG’s dominance (about 80% of total Ig) (Terry and Fahey, 1964). Meanwhile, *IGHM* was highly expressed in naïve B cells, whereas *IGHD* was highly expressed in atMBCs (Fig. S4C). In patients with LMs, atMBCs exhibited heightened inflammatory and proliferative activity, suggesting a detrimental effect on the disease (Fig. S4D). Furthermore, a comparative analysis across groups revealed higher cMBC proportions with elevated apoptosis in the Normal group, whereas LM and PE groups showed atMBC enrichment (Fig. S4E), implicating cMBC/atMBC equilibrium in sustaining immune homeostasis. We further explored the differential expression of representative functional genes of B cells. *FCRL5*, a context-dependent regulator of lymphocyte activation (Franco et al., 2018), was significantly downregulated in the PE group (Fig. S4F). This reduction correlated with diminished atMBC activity and immunocompetence, supporting an activating role of *FCRL5* in these cells (Fig. S4F and S4G). Additionally, chemokine receptors *CXCR4* and *CCR7* were upregulated in PE, aligning with enhanced chemokine signaling pathway activity (Fig. S4H and S4I).

Subsequently, we employed scBCR-seq to assess B-cell clonal expansion, while BCR expression was not significantly different among B-cell subtypes (Fig. S4J and Methods). We ranked clonotypes by their frequency of occurrence and found that the proportion of the most frequently used clonotypes increased in LMs, indicative of antigen-driven clonal expansion (Fig. S4K). Additionally, the relative proportion of clones originating from the initial node further illustrated the close relationship between cMBCs and atMBCs (Fig. S4L). In summary, pathogenic atMBCs with pro-inflammatory features underwent abnormal expansion in LMs, suggesting their dysfunction may impair B-cell-mediated immune responses.

### LMs remodel CD4^+^ T-cell composition toward pro-inflammatory and immunosuppression phenotypes

A marked reduction in CD4^+^ T-cell proportions was observed in LMs (Fig. 1F). To further investigate transcriptomic reprogramming, we focused on immune-related DEGs between the Normal and the LM groups (Fig. S5A). Downregulation of naïve/quiescent markers (such as *TCF7*, *KLF2*, and *HLA-G*) in the LM group suggested enhanced CD4^+^ T-cell activation and maturation (Fig. S5A and S5B). Furthermore, correlation analysis revealed coordinated regulation of these genes in potentiating immunoreactivity (Fig. S5C). In order to elucidate the relationships between different CD4^+^ T-cell clusters and their functions, we performed unsupervised clustering based on classical markers and categorized the cells into seven subtypes including naïve (Tn), memory (Tm), effector memory (Tem), regulatory (Treg), T helper (Th) and proliferation (T prolife) T cells (Fig. S5D and S5E). Referencing a previous study (Chu et al., 2023), we next assessed their functions using GSEA (Fig. S5F), which further supported the cluster annotation.

The composition of CD4^+^ T cells varied significantly across groups (Fig. S5G). Naïve CD4^+^ T cells (c01 and c02) were highly abundant in the Normal group, while mature CD4^+^ T cells (c03–c06) accumulated in the LM and the PE groups. Notably, CD74^+^ Th cells exhibited high inflammatory response to antigenic stimulus activity (Fig. S5F) and exhibited increased pro-inflammatory and immunoreactive characteristics (Fig. S5H). MKI67^+^ T prolife cells exhibited DNA replication pathway enrichment in LMs, indicative of disease-associated proliferative reprogramming (Fig. S5I).

We analyzed the transcriptomic dynamics of CD4^+^ T-cell activation in the LMs by reconstructing the trajectories (Fig. S5J). Naïve CD4^+^ T cells differentiated into two branches along the trajectory of development: GZMA^+^ Tem exhibiting immune effector functions, and FOXP3^+^ Treg with immunosuppressive function. Moreover, differential expression analysis identified 190 dynamically regulated genes (Fig. S5K; adjusted *P*-value < 0.05), including several immune regulators during the trajectory. For example, *DDIT4* (Miao et al., 2021) (regulating cell growth, proliferation and survival), *KLF2* (Sebzda et al., 2008) (maintaining T-cell resting state), *IL16* (Cruikshank et al., 2000) (regulating T-cell chemotaxis and cell activation) and *CTLA4* (Krummel and Allison, 1996) (controlling T-cell activation and tolerance), were disrupted in the disease group. Consistent with previous reports, our findings imply that conserved molecular alterations exist across different types of LMs.

### Transcriptional dysregulation disrupts CD8^+^ T-cell effector differentiation trajectories in LMs

**Figure 4.**
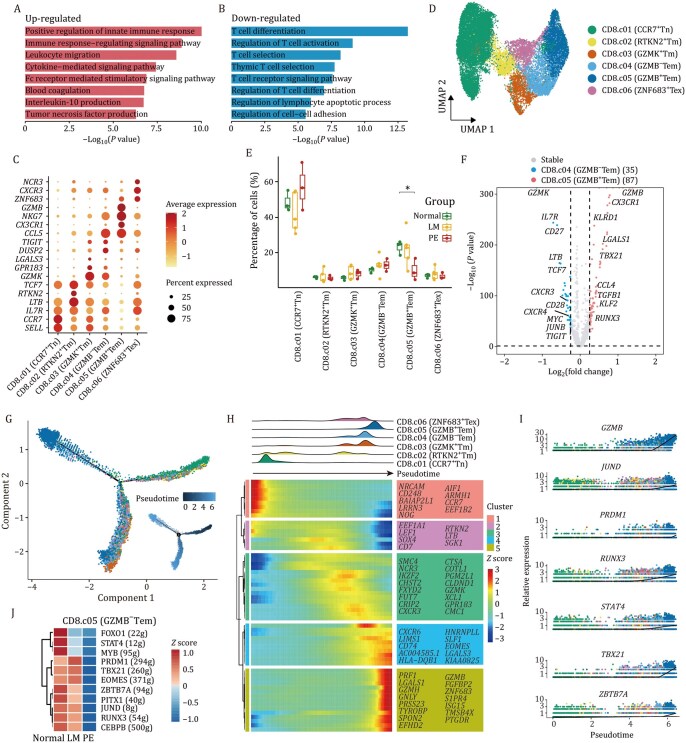
**CD8^+^ T-cell-specific dysregulation of cytotoxic function in LMs**. (A and B) Bar plot showing functional enrichment of upregulated (A) and downregulated (B) DEGs in CD8^+^ T cells, comparing the Normal group with the LM group. (C) Dotplot showing the expression of the marker genes in each cell type. (D) UMAP plot showing six CD8^+^ T-cell subtypes identified by unsupervised re-clustering. (E) Box plots showing the percentage of each CD8^+^ T-cell subtype across the three groups. Pairwise comparisons between groups were performed using the Wilcoxon test. (F) Volcano plot displaying DEGs between GZMB^+^ Tem cells and GZMB^−^ Tem cells. (G) Pseudotime analysis of the CD8^+^ T-cell lineage. Points are colored by cell types (upper) and pseudotime (bottom). (H) Ridge plot (upper) illustrating the distribution of CD8^+^ T-cell subtypes along pseudotime, colored according to cell type. Heatmap (bottom) showing pseudotime-ordered expression patterns of functionally grouped gene modules. (I) Smoothed expression curves of developmental-related genes along the pseudotime trajectory. (J) Heatmap showing the transcriptional activity of selected TFs across the three groups. *P < 0.05.

Similar to CD4^+^ T cells, the proportion of CD8^+^ T cells in LMs was also significantly reduced (Fig. 1F). GO enrichment analysis revealed upregulated immune response regulation and cytokine production, contrasted by downregulated T-cell differentiation/activation (Fig. 4A and 4B), suggesting that CD8^+^ T cells in LMs responded actively to external stimuli with compromised maturation. Furthermore, unsupervised clustering analysis identified 12 clusters of CD8^+^ T cells, with each cluster present across multiple samples (Fig. S6A and S6B). Based on canonical markers and curated gene signatures (Fig. 4C), we defined six transcriptional states: naïve (Tn, c01), memory (Tm, c02 and c03), effector memory (Tem, c04 and c05) and exhausted (Tex, c06) subtypes (Fig. 4D). Genes such as *PRF1*, *NKG7*, and *FGFBP2* were all highly expressed in GZMB^+^ Tem cells (Figs. 4C and S6C), indicating a potent cytotoxic ability in these cells. This was further supported by functional enrichment and scoring analysis (Fig. S6D and S6E).

Notably, two effector memory subtypes exhibited distinct distributions: GZMB^+^ Tem cells were enriched in the Normal group, while GZMB^−^ Tem cells accumulated in the LM and PE groups (Fig. 4E). Differential analysis between them revealed the former possessed significantly higher toxicity and killing ability (Fig. 4F; Table S5), further supported by TCR clonal similarity analysis (Fig. S7E). To explore the developmental trajectories among CD8^+^ T-cell subtypes, we initially conducted a trajectory analysis. Naïve CD8^+^ T cells differentiated into two branches (Fig. 4G): GZMB^+^ Tem cells (representing an effector cytotoxic state) and ZNF683^+^ Tex cells (indicating an exhausted state), with GZMB^−^ Tem serving as an intermediate differentiation stage. A list of potential key genes implicated in the transition toward an effector state of CD8^+^ T cells was compiled, drawing on multiple lines of evidence from independent analyses (Fig. 4H). Several of these genes, such as *JUND*, *RUNX3*, and *TBX21* (Fig. 4I), have previously been linked to the cell development process. Moreover, TF analysis uncovered master regulators in effector memory subtypes with strong regulatory activity (Fig. S6F). In GZMB^−^ Tem cells, the regulatory activity or gene expression of these TFs was decreased in LM patients (Figs. 4J and S6G), indicating aberrant transcriptional regulation along the trajectory toward the GZMB^+^ Tem state. Taken together, these findings provide insights into how abnormal expression or activity of TFs may disrupt the differentiation trajectories between two effector memory subtypes in disease state, thereby affecting cytotoxicity of CD8^+^ T cells.

### Heterogeneous features in clonality and diversity of TCRs associated with LMs

**Figure 5.**
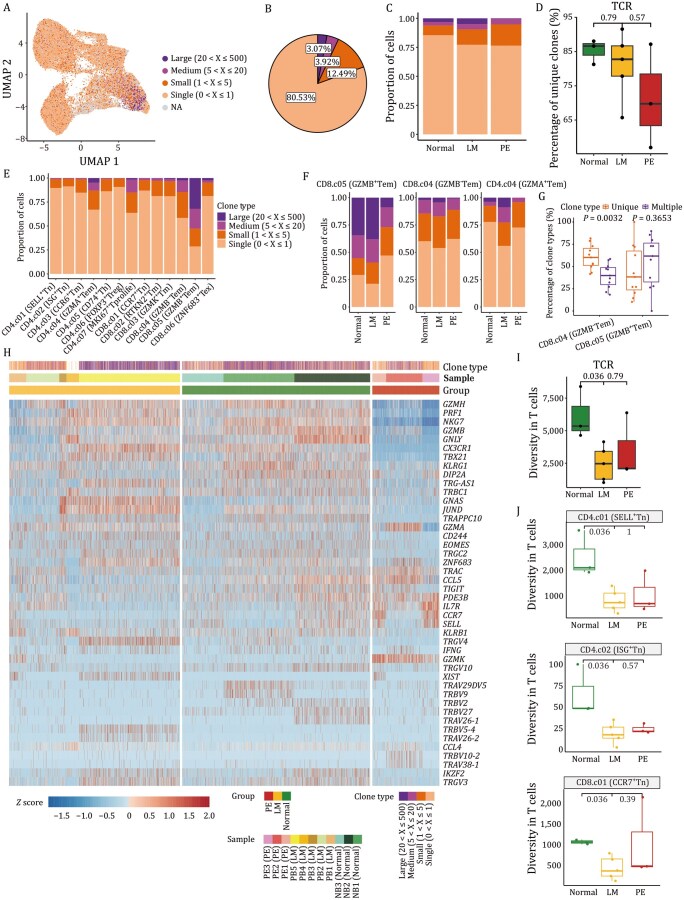
**Characterization of TCR landscape and dynamics in T-cell subtypes**. (A) UMAP visualization of T-cell subtypes integrated with TCR clonality. Colors indicate clonotype frequency groups. X represents the frequency of each clonotype defined by its unique paired TCRαβ sequence. (B) Pie plot showing the percentage of each TCR clone type among all T cells. (C) Bar plot showing the percentage of each TCR clone type across the three groups. (D) Box plot showing the percentage of unique clones across the three groups. Each dot represents one sample, two-sided Wilcoxon rank-sum tests. (E) Bar plot showing percentage of clonal lineages categorized by clonal size in each TCR clone type. (F) Bar plots showing the percentage of each TCR clone type in CD8.c05 (GZMB^+^ Tem), CD8.c04 (GZMB^−^ Tem) and CD4.c04 (GZMA^+^ Tem) subtypes across the three groups. (G) Boxplots showing the percentage of TCR clones in CD8.c04 (GZMB^−^ Tem) and CD8.c05 (GZMB^+^ Tem) subtypes between unique and multiple clone types. Each dot represents one sample, two-sided Wilcoxon rank-sum tests. (H) Heatmap illustrating single-cell expression of selected genes (rows). Expression was *Z*-score normalized per gene across all cells. Each column is a single T cell; cells are grouped by clonal type, sample, and group. Individual T cells are ordered by clonal size. (I) Box plot showing diversity of TCR across the three groups. Each dot represents one sample, two-sided Wilcoxon rank-sum tests. (J) Box plots showing TCR diversity in CD4.c01 (SELL^+^ Tn), CD4.c02 (ISG^+^ Tn) and CD8.c01 (CCR7^+^ Tn) subtypes across the three groups. Each dot represents one sample, two-sided Wilcoxon rank-sum tests.

By analyzing the TCR repertoire, which plays a crucial role in antigen recognition and T-cell activation, we can gain precise insights into T-cell development, expansion, and differentiation (Bradley and Thomas, 2019). We analyzed the TCR repertoire using integrated scRNA-seq and scTCR-seq data from 13 T-cell subtypes (seven CD4^+^ T-cell subtypes and six CD8^+^ T-cell subtypes) (Fig. S7A, S7B and Methods). Expanded clonotypes were defined based on the expression of identical paired TCRα and TCRβ chains by two or more T cells (Moon et al., 2023). From approximately 30,000 TCRαβ pairs obtained, we identified a range of clonal lineage sizes, including large (clonal lineage members, X > 20), medium (5 < X ≤ 20) and small (1 < X ≤ 5) lineages (Fig. 5A). Overall, clonally expanded T cells constituted approximately 20% of TCR^+^ cells (Fig. 5B), with significant enrichment in LMs (Fig. 5C and 5D), indicating antigen-driven clonal expansion of T cells. In addition, the clonally expanded T cells were predominantly located in effector subtypes: CD4.c04 (GZMA^+^ Tem), CD8.c04 (GZMB^−^ Tem), and CD8.c05 (GZMB^+^ Tem), particularly CD8.c05 (GZMB^+^ Tem) (Fig. 5E), where the proportion of expanded clones was significantly higher in LMs than in Normal (Fig. 5F and 5G). These clonally expanded CD8^+^ T cells expressed cytotoxic markers (e.g., *GZMH*, *GZMB*) and migratory markers (e.g., *CX3CR1*, *KLRC1*) (Fig. 5H).

Clonality and diversity are often used as measures of immune response efficacy (Porciello et al., 2022). Naïve T-cell subtypes, including CD4.c01 (SELL^+^ Tn), CD4.c02 (ISG^+^ Tn), and CD8.c01 (CCR7^+^ Tn), exhibited the highest TCR diversity (Fig. S7C and S7D), which were reduced in LMs (Fig. 5I). Notably, naïve T-cell subtypes exhibited decreased diversity in the LM and PE groups (Fig. 5J), reflecting impaired immune responsiveness. In summary, LMs are characterized by increased T-cell clonality, decreased naïve T-cell diversity.

### Transcriptional dysregulation in NK cells mediates the reduced cytotoxic capacity

**Figure 6.**
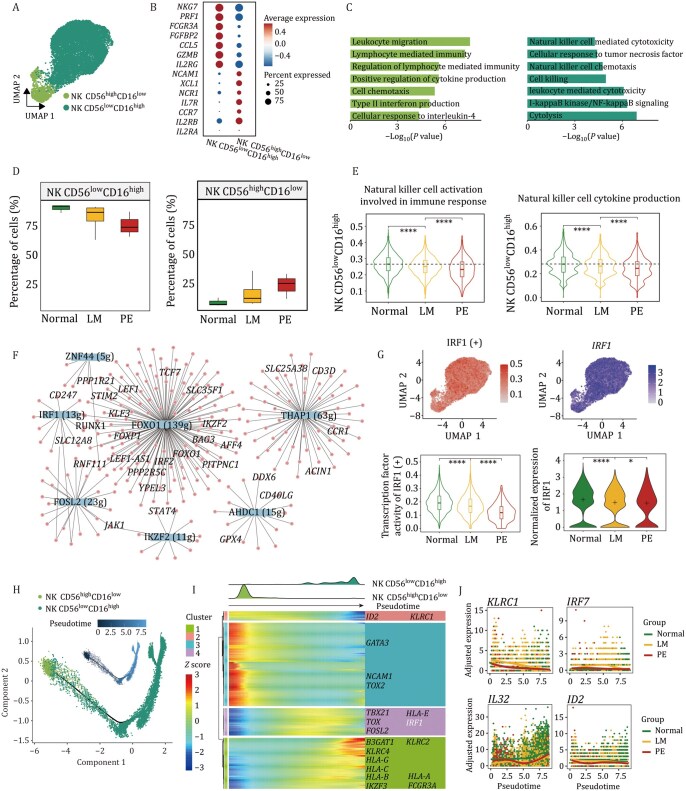
**NK cell-specific immune dysregulation during pathological remodeling in LMs**. (A) UMAP plot showing two NK cell subtypes identified by unsupervised re-clustering. (B) Expression patterns of regulatory and cytotoxic gene signatures in CD56^low^CD16^high^ and CD56^high^CD16^low^ NK cells. (C) Bar plots showing representative biological functions enriched in CD56^high^CD16^low^ (left) and CD56^low^CD16^high^ (right) NK cells. (D) Box plots displaying the proportion of two NK cell subtypes across the three groups. (E) Violin plots showing the scores of selected pathways in CD56^low^CD16^high^ NK cells across the three groups, two-sided Wilcoxon rank-sum tests. (F) Regulatory network visualization of potential transcriptional regulators in NK cells. Larger nodes represent TFs, and smaller nodes represent their target genes. (G) UMAP plots (upper) showing IRF1 transcription factor activity and its gene expression distribution. Violin plots (bottom) showing IRF1 activity and gene expression across the three groups, two-sided Wilcoxon rank-sum tests. (H) Pseudotime trajectory of NK cell lineage. Points are colored by pseudotime (upper) and by cell types (bottom). (I) Ridge plot (upper) illustrating the distribution of NK cell subtypes along pseudotime. Heatmap (bottom) depicting the expression patterns of 216 genes that exhibit significantly different expression patterns along the cell differentiation trajectory. The significance threshold was set as an adjusted *P*-value < 0.05. (J) Smoothed expression curves of representative candidate genes along the trajectory. *P < 0.05, ****P < 0.0001.

NK cells, key regulators of immune surveillance, orchestrate disease control through direct cytotoxicity and pro-inflammatory cytokine secretion (Freud and Caligiuri, 2006; Huntington et al., 2007). Here, NK cells were subdivided into two subtypes (CD56^low^CD16^high^ and CD56^high^CD16^low^) based on the expression levels of CD56 (*NCAM1*) and CD16 (*FCGR3A*) (Lanier et al., 1986) (Figs. 6A and S8A). The CD56^low^CD16^high^ NK cell population predominantly mediates the killing of target cells by secreting interferon and granzymes, whereas the CD56^high^CD16^low^ NK cell population exhibits immunoregulatory and cytokine-producing capacities (Fig. 6B and 6C). We next assessed the preference of NK cell population among different groups and observed clear discrepancies (Fig. S8B). The CD56^high^CD16^low^ NK cells were largely expanded in LMs, whereas the proportion of CD56^low^CD16^high^ NK cells was decreased (Fig. 6D), consistent with an increase in inflammation and a decrease in immune function (Figs. 6E and S8C).

To elucidate the upstream mechanisms underlying the reduced proportion and diminished cytotoxicity of NK cells, we predicted potential TFs. The results unveiled dynamic changes in the transcriptional activity of those TFs between the two subtypes (Fig. S8D), which regulated a lot of target genes involved in immune cell differentiation, as depicted in the network topology (Figs. 6F and S8E). Among these factors, the transcriptional activity and gene expression level of IRF1 (Mamane et al., 1999; Taniguchi et al., 2001) (a key TF downstream of the pathogen recognition signaling pathway, regulating the IFN-I (IFNα/β) response and related antiviral immune responses) were significantly reduced (Figs. 6G and S8F). This reduction potentially mediated the weakened cytotoxic and immunoregulatory responses observed in LMs.

Furthermore, we revealed that CD56^low^CD16^high^ NK cells were at a late stage of differentiation (Fig. 6H). TFs such as *ID2* and *GATA3* primarily governed the early stages of NK cell development, while *IRF1*, *TBX21*, and *TOX* exerted stronger regulatory influence during the intermediate stages, and *B3GAT1* and *IKZF3* participated in the late-stage regulation (Fig. 6I). Additionally, we observed alterations in the expression trajectories of certain genes in the context of LMs (Fig. 6J). For instance, *KLRC1* (also known as *NKG2A*, an “inhibitory” receptor for NK cell activity) and *IRF7* (a key regulator of type I interferon signaling) exhibited abnormal upregulation in the early stages of disease progression. Conversely, the expression levels of *IL32* (which can activate NK cell cytotoxicity through *DR3*) and *ID2* (a factor associated with NK cell maturation) were reduced, indicating a disruption in the NK cell developmental process during disease progression. Taken together, the dysregulation in the expression and activity of TFs in LMs may disrupt the developmental trajectory, consequently exerting a pronounced impact on the functional potential of NK cells.

### Immune cell communication remodeling in the LMs microenvironment

**Figure 7.**
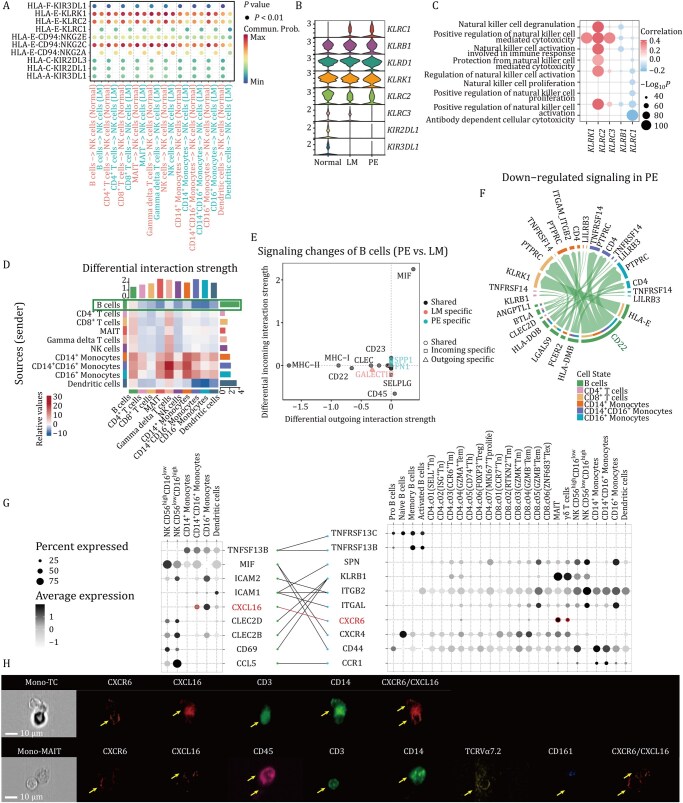
**Disrupted intercellular communication mediates the impaired cytotoxic activity and pro-inflammation of immune cells**. (A) Comparison of significant ligand–receptor pairs between the Normal and LM groups that contribute to the signaling of NK cells. Dot color represents communication probability, and dot size indicates the corresponding *P*-value. Empty spaces indicate zero communication probability. *P*-values are computed from one-sided permutation test. (B) Violin plots showing the expression patterns of genes involved in NK cell activity-related signaling. (C) Bubble plot showing correlations between receptor gene expression and pathway activity. Pearson correlation coefficients and two-tailed *P* values. (D) Heatmap showing differential interaction strength between the Normal and LM groups. (E) Dot plot showing signaling changes of B cells in the PE group compared with the LM group. (F) Circos plot showing downregulated receptors and ligands mediating interactions between B cells and other cell types in the PE group compared with the LM group. (G) Dot plot showing the expression of inflammation-related ligands (left) and receptors (right) across immune cells. Only significant LR interactions are connected by lines. (H) Interactions between monocytes and T cells (top) or MAIT cells (bottom) mediated by CXCL16–CXCR6 were analyzed. Images show expression of CD45, CD14, CD3, CD161, TCRVα7.2, CXCR6, CXCL16. Imaging flow cytometry data were derived from six subjects across three independent experiments; representative images are shown; Scale bar, 10 µm. Specific fluorescent dyes, CD45 (APC–CY7), CD14 (BV510), CD3 (FITC), CD161 (BV421), TCRVα7.2 (PE), CXCR6 (RB780), CXCL16 (BV605).

Given the importance of immune cell communication in maintaining immune homeostasis, we first examined immune cell interactions using known ligand–receptor (LR) pairs. Although we did not observe significant differences in the overall number or intensity of interactions across groups (Fig. S9A–C and Methods), a more granular analysis revealed notable cell type-specific alterations. Compared with the normal group, CD4^+^ T cells exhibited increased communication with multiple immune cell types, whereas CD8^+^ T-cell-mediated interactions were diminished (Fig. S9D). To further dissect immune-regulatory circuitry, we characterized immune checkpoint-associated LR interactions (Fig. 7A). Several co-inhibitory (HLA-E–CD94:NKG2A) and co-stimulatory (HLA-E–CD94:NKG2C and HLA-E–CD94:KLRC2) pairs were identified between NK cells and other immune cells. Notably, the inhibitory receptor NKG2A (encoded by *KLRC1*) was selectively upregulated in NK cells from LMs patients (Fig. 7B), while the activating receptors NKG2C (encoded by *KLRC2*) and NKG2D (encoded by *KLRK1*) were reduced. Those genes were positively correlated with the cytotoxicity of NK cells (Fig. 7C), suggesting impaired antiviral responses and highlighting a potential immunotherapeutic strategy for LMs.

We next compared interaction strengths across the Normal, LM, and PE groups and found that B cells showed markedly decreased outgoing interaction strength in LMs—most prominently in the PE group (Fig. S9E–G). Correspondingly, B cells acting as signal senders exhibited reduced communication with multiple immune populations (Figs. 7D and S9H). Many of the diminished signals in the PE group involved immune-regulatory pathways, including MHC-II, MHC-I, CD22, and CLEC signaling (Figs. 7E and S9I). CD22, which is highly expressed on atMBCs (Fig. S9J) and functions as a negative regulator to prevent excessive B-cell activation, showed reduced signaling between B cells and myeloid cells (Fig. 7F). This finding suggested impaired CD22-mediated inhibition, potentially leading to overactivation of B-cell–monocyte communication in the microenvironment. CXCR6^+^CD8^+^ T cells, with cytotoxicity and proliferation potential, were highly enriched in the inflammatory tissue, such as synovial fluid from patients with inflammatory arthritis (Kim et al., 2001) and colon in Crohn’s disease (Mandai et al., 2013), implying CXCR6 might play a critical role in pathogenic T-cell migration into inflammatory tissue in autoimmune disease. Through communication analysis (Fig. 7G; Table S6), we identified that CD14^+^CD16^+^ monocytes may play a pro-inflammatory role through CXCL16 binding to CXCR6-expressing MAIT or γδ T cells. Using imaging flow cytometry (Fig. 7H and Methods), we specifically confirmed CXCL16 expression on monocytes and its interaction with CXCR6-expressing MAIT and T cells in LM patients. Taken together, we profile the immunocyte interactions in blood and PE at a single-cell scale and reveale the impact of immune checkpoint molecules on cellular activity and function.

### A shared pro-inflammatory monocyte program reveals therapeutic targets in LMs

**Figure 8.**
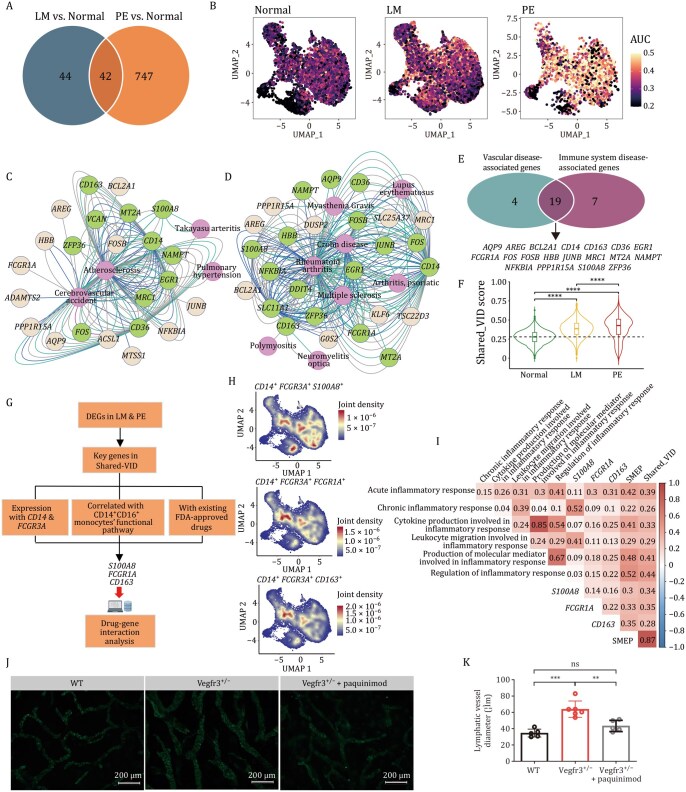
** Identification of candidate FDA-approved drugs for the treatment of LMs**. (A) Venn plot illustrating 42 co-upregulated genes shared by LM vs. Normal and PE vs. Normal in CD14^+^CD16^+^ monocytes. (B) Feature plots showing the expression pattern of SMEP across the three groups. (C and D) Network visualization of disease-gene interaction maps of SMEP-related vascular (C) and immune system (D) diseases. Genes are colored in beige and those associated with at least two diseases are colored in green; diseases are colored in purple. Network is built using prefuse force-directed layout algorithm. (E) Venn plot (upper) showing the SMEP-derived 19 genes that are involved in both vascular and immune system diseases. (F) Violin plot showing the Shared-VID score across the three groups, two-sided Wilcoxon rank-sum tests. (G) Schematic representation of the drug target selection criteria for drug–gene interaction analysis. (H) Co-expression visualization of *S100A8* (upper), *FCGR1A* (middle), and *CD163* (bottom) with CD14^+^CD16^+^ monocyte markers (*CD14* and *FCGR3A*) in myeloid cells. (I) Heatmap showing gene and pathway correlation analysis of the drug targets, *S100A8*, *FCGR1A*, and *CD163*, with SMEP, Shared-VID, and selected pathways. Pearson correlation coefficients and two-tailed *P*-values. (J) Immunofluorescence images of lymphatic vessels in mouse ear tissue in WT mice (left), Vegfr3^+/−^ mice (middle), and Vegfr3^+/−^ mice with paquinimod treatment (right); Scale bar, 200 µm. (K) Quantification of lymphatic vessel diameter in three groups of mice ear tissue. Mean ± SEM, unpaired *t*-test. WT mice (*n *= 5), Vegfr3^+/−^ mice (*n *= 6), and Vegfr3^+/−^ mice with paquinimod treatment (*n *= 5). **P < 0.01, ***P < 0.001, ****P < 0.0001.

To dissect the molecular mechanisms underlying the enhanced pro-inflammatory activity of CD14^+^CD16^+^ monocytes, we identified a shared pathological signature between the LM and PE groups. Differential expression analysis comparing LM/PE groups to the Normal group revealed an SMEP consisting of 42 upregulated DEGs (Figs. 8A and S10A), suggesting potential targets for therapeutic intervention. The SMEP was significantly activated in both disease states, with notable overexpression of genes (Figs. 8B and S10B), such as *NFKBIA* (associated with the NF-κB pathway), *S100A8* (mediated intracellular inflammatory signal transduction), *FCGR1A* (involved in phagocytosis, degranulation, and pro-inflammatory cytokine production), and *CD163* (involved in the regulation of inflammatory process), which are pivotal in inflammatory processes and immune responses (Fig. S10C). Pathway enrichment analysis showed the SMEP was associated with inflammation, immune cell activation, and stress-response pathways (Fig. S10D), underscoring the contribution of CD14^+^CD16^+^ monocytes to LM pathogenesis.

Given that elevated intermediate monocytes have been reported in stroke, rheumatoid arthritis (RA), and nephritis (Narasimhan et al., 2019; Patel et al., 2017), we next investigated the relevance of the SMEP to vascular and immune disorders. Using Disease Gene Network (DisGeNET), we mapped SMEP genes to disease categories (i) vascular diseases and (ii) immune system disorders (Figs. 8C, 8D and S10E; Table S7 and Methods), and found that 23 genes were associated with vascular diseases, 26 genes were mapped to immunity disorders, and 19 genes (defined as “Shared-VID”) were associated with both (Fig. 8E). The Shared-VID signature of CD14^+^CD16^+^ monocytes was consistently upregulated in the LM and PE groups (Fig. 8F), and enriched in pathways related to inflammatory regulation, oxidative stress response, and response to biotic stimuli (Fig. S10F), supporting their pathological relevance in LMs.

Leveraging the SMEP, we further screened for potential therapeutic strategies by querying FDA-approved drugs that target SMEP-associated genes (Fig. 8G; Table S8 and Methods). From 19 DGIdb categories, we found 16 genes involved in SMEP, with FDA-approved drugs, and eventually focused on three targets: *S100A8*, *FCGR1A*, and *CD163*, based on their co-expression with CD14^+^CD16^+^ monocytes markers (*CD14* and *FCGR3A*) (Fig. 8H). Additionally, their expression levels were upregulated in both the LM and PE groups (Fig. S10G). Pearson correlation analysis further supported their involvement in disease-modifying inflammatory pathways (Fig. 8I). A total of 50 FDA-approved drugs could potentially interact with the 16 targets (Fig. S10H), including drugs such as fluticasone (used for asthma and rhinitis) and methotrexate (used for RA). Our single-cell analysis illuminates potential targets and drugs for LMs, providing a foundation for future clinical exploration and the potential repurposing of existing FDA-approved therapies.

### Paquinimod significantly ameliorated the lymphangiogenic phenotype in Vegfr3^+/−^ mice

The main drivers of lymphangiogenesis and pathological lymphangiogenesis are VEGFR3 and its secretory ligand VEGFC (Monaghan et al., 2024). In particular, Vegfr3-CreER^T2^ recombinase-mediated activation of the frequent causative mutation in LECs of transgenic mice was shown to promote lymphatic vessel overgrowth and formation of vascular lesions in mice (Makinen et al., 2021; Martinez-Corral et al., 2020; Rodriguez-Laguna et al., 2019). Sequentially, we constructed *Vegfr3* knockdown mice to evaluate the effect of the above-mentioned target on LMs (Methods).

S100A8 belongs to the S100 calcium-binding protein family and has recently gained much interest as a critical alarmin modulating the inflammatory response after releasing from neutrophils and monocytes (Feng et al., 2023; Pruenster et al., 2016; Silvin et al., 2020). We next explored whether S100A8 has a potential therapeutic value in LMs. Paquinimod (TahviLi et al., 2018) is a selective inhibitor of S100A8/A9 by preventing S100A8/A9 binding to TLR4. WT mice and Vegfr3^+/−^ mice were administered with vehicle or paquinimod for 4 weeks (Fig. 8J). We found the phenotype of the LMs was ameliorated by paquinimod treatment in Vegfr3^+/−^ mice (Fig. 8K). Taken together, our study suggests S100A8 as a potential target for treating LMs.

### The unique phenotypic characteristics of immune cells in LMs

LMs are increasingly recognized to involve immune dysregulation, yet their immunological distinctions from classical immune-mediated diseases remain elusive. To address this, we collected and profiled 188,974 single cells from four diseases (LMs, nephritis [An and Duan, 2022], stroke [Goods et al., 2021], and RA [Sonigra et al., 2022]) and two healthy control groups (peripheral blood from adults and children [Liu et al., 2022]) after quality control (Fig. S11A; Table S9). Following data integration across samples via Harmony and subsequent manifold construction, we annotated 11 major immune cell types based on canonical marker genes (Fig. S11B and S11C). By comparing immune characteristics across the four diseases using their highly expressed gene signatures, we found that T cells in LMs exhibited pronounced activation, especially in receptor signaling pathways (Fig. S11D). In monocytes, LMs displayed enhanced phagocytic activity and inflammatory responses, whereas RA was characterized by macroautophagy and cytokine production (Fig. S11E). Our analysis further focused on the disease differences across the four groups and discovered numerous distinct DEGs in LMs (Fig. S11F). For instance, key inflammatory mediators (*S100A4*, *GZMA*, *S100A11*, *EGR1*, *FOS*, and *FOSB*) were specifically upregulated in LMs, whereas antigen presentation genes (*HLA-DRB5* and *HLA-G*) were downregulated. Intriguingly, NK cells in LMs showed selective upregulation of *KLRC1* (encoding inhibitory receptor NKG2A), indicative of impaired cytotoxic function. Furthermore, elevated *BCL2* expression in CD4^+^/CD8^+^ T cells correlated with lymphoma-associated T-cell survival dysregulation (Feng et al., 2010). We subsequently observed a pro-inflammation state in LMs, characterized by elevated interferon production in B cells and CD14^+^ monocytes, alongside diminished immune tolerance induction across multiple lymphoid cell types (Fig. S11G). Notably, the antigen processing and presentation capacity of CD14^+^ monocytes was downregulated among the four groups (Fig. S11G), especially in LMs, which was crucial for maintaining homeostasis and pathogen defense. Collectively, these findings delineate a unique immunopathological landscape in LMs, characterized by pervasive hyperactivation, inflammatory dysregulation, and impaired antigen presentation, distinguishing it from other inflammatory diseases and underscoring its distinct mechanistic underpinnings.

## Discussion

LMs are rare and severe diseases of largely unknown etiology (Makinen et al., 2021; Zhong et al., 2021), associated with impaired interstitial fluid drainage, immune dysfunction, and nutritional deficiencies (Itkin et al., 2020). Despite these challenges, the immune cell signature in LMs remains poorly delineated. Here, we constructed a ­comprehensive single-cell atlas of immune cells from peripheral blood and PE in LMs, revealing heightened inflammation and immune dysregulation.

Consistent with clinical findings, blood samples from LMs exhibited an increased proportion of B cells and myeloid cells, associated with heightened inflammatory responses. In contrast, PE exhibited the enrichment of CD8^+^ T cells and the reduction in B cells and myeloid cells. These findings provide detailed information on immune cell types and their potential impact on the progression of LMs. Previous studies (Aebischer et al., 2014; Baluk et al., 2005) have revealed that inflammation induces rapid, stimulus-specific changes in LEC gene expression and a stimulus-specific expansion and remodeling of the lymphatic network. We further identified multiple pro-inflammatory subtypes in LMs, which regulate inflammatory cytokine production and influence lymphatic expansion and lymphedema. Intriguingly, CD14^+^ monocytes appeared to downregulate the expression of MHC molecules, suggesting a potential impairment in antigen presentation and processing functions, as preliminarily observed in our *in vitro* functional assays.

Cytolytic effector CD8^+^ T cells eliminate pathogens through cytokine secretion and direct killing of infected cells. However, in LMs, GZMB^+^CD8^+^ T cells, which are crucial for cytotoxicity, were reduced, indicating impaired cytotoxicity and killing function. Recent studies suggest that the environmental adaptations of CD8^+^ T cells are intertwined with their developmental trajectories, which determine their effector responses and long-term immunity (Reina-Campos et al., 2021). We demonstrated that GZMB^−^ CD8^+^ T cells can differentiate into GZMB^+^ CD8^+^ T cells, providing a new insight into the functional diversification and plasticity of CD8^+^ T-cell responses. Over the past decade, several TFs (EOMES, T-BET, RUNX3, CEBPB, and FOXO1) (Kaech and Cui, 2012; Zitti et al., 2023) have been identified as key regulators of CD8^+^ T-cell effector and memory development. Our trajectory analysis revealed dynamic changes in the expression of these factors, highlighting the need to further elucidate the upstream regulatory mechanisms driving CD8^+^ T-cell states in LMs.

CD8^+^ T cells and NK cells are both cytotoxic effector lymphocytes, yet they differ substantially in terms of antigen recognition, specificity, and memory formation. Although CD8^+^ T cells have been extensively characterized, the biology of NK cells in LMs remains less understood. In LMs, we observed a reduced frequency of CD56^low^CD16^high^ NK cells and diminished cytotoxic activity. This functional decline may be linked to diminished expression and regulatory activity of key TFs (e.g., IRF1, ID2), potentially disrupting NK cell maturation. NK cell cytotoxicity is tightly regulated by a balance of activating and inhibiting signals. Notably, the inhibitory receptor NKG2A, which is upregulated in settings of chronic antigen exposure and contributes to tumor immune evasion (Borst et al., 2020; Salome et al., 2022), was also found to be elevated on NK cells in LMs. This finding suggests that the upregulation of *NKG2A* on NK cells in LMs may impair their cytotoxic function. Therefore, we suggest that *NKG2A* blockade, either alone or together with other checkpoint inhibitors, might improve the efficacy of NK and CD8^+^ T cells in LMs.

Previous studies (Bao et al., 2023; Piehl et al., 2022; Wang et al., 2024) indicated that CXCR6 plays a pivotal role in regulating inflammation and tissue damage through its interaction with CXCL16. In our dataset, we observed evidence of CXCL16‑expressing monocytes interacting with CXCR6‑positive MAIT and γδ T cells. We further validated this interaction in the context of LMs using imaging flow cytometry. These findings suggest that the CXCL16–CXCR6 axis may also contribute to the inflammatory microenvironment in LMs, warranting further functional investigation.

We identified an SMEP in LMs, reflecting a common pathological mechanism. This program was activated in both the LM and PE groups, involving key genes in inflammation and immune responses. By integrating the SMEP with GWAS data, we further identified risk genes associated with vascular and immune diseases, which helped prioritize potential therapeutic targets. Therefore, by analyzing drug–gene interactions of the SMEP, we identified FDA-approved drugs that may inhibit CD14^+^CD16^+^ monocytes hyperactivation by targeting *S100A8*, *FCGR1A*, or *CD163*. Among these, *S100A8* has garnered increasing attention for its pleiotropic roles in inflammation, immune modulation, and tissue repair (He et al., 2025; Li et al., 2019; Silvin et al., 2020). To functionally validate the therapeutic relevance of disrupting this pathway, we administered paquinimod (an S100A8/A9 inhibitor) in a Vegfr3^+/−^ mouse model of LMs, and observed significant amelioration of the malformation phenotype. These results collectively nominate S100A8 signaling as a potential target for clinical intervention in LMs.

In conclusion, our study delineates the immune landscape of LMs, characterizing a microenvironment marked by concomitant inflammatory activation and functional immunodeficiency. We have pinpointed specific transcriptional regulators and immune checkpoint molecules as promising candidate biomarkers and therapeutic targets. The therapeutic relevance of targeting the inflammatory axis was functionally validated in a Vegfr3^+/−^ mouse model of LMs, where administration of paquinimod, an S100A8 inhibitor, significantly ameliorated the malformation phenotype. These findings collectively nominate the S100A8 signaling pathway as a compelling target for clinical intervention in LMs. Looking forward, beyond pharmacological strategies, future investigations into advanced cellular therapeutics, such as CAR-T or CAR-NK cell therapies, may open new promising avenues for intervention.

## Supplementary Material

pwaf103_Supplementary_Data

## Data Availability

The scRNA-seq raw data and WES data have been deposited in the National Genomics Data Center (HRA006623).

## Abbreviations

CCLA,central conducting lymphatic anomaly; DEGs, differentially expressed genes; DGIdb, drug-gene interaction database; DisGeNET, disease gene network; FDA, Food and Drug Administration; GSEA, gene set enrichment analysis; LECs, lymphatic endothelial cells; LMs, lymphatic malformations; LR, ligand-receptor; MAIT, mucosal associated invariant T; pDC, plasmacytoid dendritic cells; PE, pleural effusion; RA, rheumatoid arthritis; scBCR-seq, B-cell receptor sequencing; scRNA-seq, single-cell RNA sequencing; scTCR-seq, T-cell receptor sequencing; SMEP, shared CD14^+^CD16^+^ monocyte expression program; TFs, transcription factors; WES, whole-exome sequencing

## Code availability

All custom computer code developed for analysis of scRNA-seq data in this study is available at github.com/SunLab-Bioinformatics/Lymphatic-malformations.
