# Supplementary material for: Multidimensional single-cell analysis reveals immune dysfunction and inflammatory response in lymphatic malformations
Source: Protein Cell. 2025 Nov 22;17(4):320–38. doi: 10.1093/procel/pwaf103 (PMC13107555; doi:10.1093/procel/pwaf103)
Supplement: pwaf103_Supplementary_Data [file pwaf103_supplementary_data.zip › PAC-25003-SBF-Supplementary Materials.pdf]

Supplemental Material

Methods

Study patients and sample collection

Six patients with a definite diagnosis of LMs (the specific disease type: CCLA) were enrolled in our study (Table S1), and all participants provided written informed consent. Pleural effusion samples from each subject were collected in heparin-treated tubes within 24 hours after hospitalization, using standard thoracentesis techniques. Approximately 5 mL of venous blood was drawn simultaneously. The pleural effusion specimens were immediately immersed in ice and then centrifuged at 800 × g for 5 minute. The cell-free supernatants from pleural effusion and sera were frozen at -80 °C immediately after centrifugation. Mononuclear cells from pleural effusion and blood were isolated by gradient centrifugation (Pharmacia, Uppsala, Sweden) within 1 hour and resuspended in phosphate-buffered saline.

Cell capture and single-cell sequencing

For droplet-encapsulation scRNA-seq experiments, cell suspension was loaded onto a Chromium Single Cell Controller (10x Genomics) to generate single-cell gel beads-in-emulsion (GEMs) according to the manufacturer's protocol. Captured cells were lysed and the released RNA was barcoded through reverse transcription within individual GEMs. Reverse transcription was performed on a S1000TM Touch Thermal Cycler (Bio Rad) at 53°C for 45 min, followed by 85°C for 5 min, and a hold at 4°C. The cDNA was generated and then amplified, and quality assessed using an Agilent 4200.

Libraries were generated using the Chromium Single Cell 5' Feature Barcode Library Kit (10x Genomics), Chromium Single Cell 5'Library and Gel Bead Kit (10x Genomics, 1000006) and Chromium Single Cell A Chip Kit (10x Genomics, 120236). The libraries from each channel were multiplexed together and sequenced on an Illumina NovaSeq 6000 platform. The resulting FASTQ files were processed using the Cell Ranger pipeline (version 7.1.0) aligned to the GRCh38 reference genome.

Whole exome sequencing (WES) and bioinformatics analysis

Genomic DNA was extracted from patient blood samples using the Qiagen kit. Sequencing libraries were prepared using the Hieff NGS® OnePot Pro DNA Library Prep Kit for Illumina and exome capture was performed with IDT V1 probes. Whole exome sequencing was conducted on Illumina NovaSeq instruments. Raw data underwent quality control with FastQC (version 0.11.9) and adapter trimming using Trim Galore (version 0.6.10). Cleaned reads were aligned to the human reference genome (hg38) using BWA-MEM (version 0.7.17-r1188). The Genome Analysis Toolkit (GATK, version 4.6.1.0) (DePristo et al., 2011) was used for somatic variant calling. Bioinformatic analysis included base quality score recalibration (BQSR) and somatic mutation detection with Mutect2 using a panel of normals and gnomAD as

germline resource. Variants were filtered to retain only PASS calls. All somatic variants were annotated using ANNOVAR (Wang et al., 2010) to collect gene information, functional consequences (including missense, nonsense, frameshift, and splice-site variants), population frequency from gnomAD211_exome, and cancer association from COSMIC70. Variants were further filtered to retain those with gnomAD allele frequency < 0.001 or absent in population databases.

Processing of scRNA-seq data with the Seurat package and quality control

To reduce noise in the data, we performed a two-run clustering. Examining the result from the first run clustering, we identified contamination clusters and clusters that arose from unwanted factors. The second run clustering without such noise prepared the basis for data integration across multiple datasets. The detailed steps are as follows:

The Seurat package (version 4.3.0) (Hao et al., 2021) was used for quality control. Cells with fewer than 200 genes detected or > 7% mitochondrial unique molecular identifier (UMI) counts were filtered out; genes detected in > 3 cells were kept. Furthermore, we normalized the data using the R package *SCTransform* (version 0.4.0, vars.to.regress = c("nCount_RNA", "orig.ident", "percent.mt")). Then we ran the Seurat pipeline for the first time: We performed principal component analysis (PCA) using the top 2,000 highly variable genes (HVGs) (excluding genes in the blacklist (Zheng et al., 2021), Table S10) identified by the *FindVariableFeatures* function with the selection method as vst (Stuart et al., 2019); Clustering with 20 principal components (PCs) and a resolution of 1.5 resulted in 43 clusters (clusters 0–42). We found that a few clusters exhibited low quality characteristic, including low UMI counts, high percentages of mitochondrial UMI counts, or the dissociation-related score or no conventional marker genes). The potential contaminations or doublet cells were removed by dropping the clusters or by filtering out cells with high average expression of the signature genes from contaminating cell types. After filtering, 92,088 cells remained across all samples.

Next, we ran the Seurat pipeline for the second time: same as the first run, we selected the top 2,000 HVGs, used 20 PCs and a resolution of 1.5 to perform the second-round clustering and annotated each cluster based on canonical marker genes. We identified 14 major cell types, including seven lymphoid cell types and seven myeloid cell types (Fig. 1C - 1E; Table S3).

Sequencing and analysis of TCR and BCR V(D)J

Full-length TCR/BCR V(D)J segments for the amplified cDNA from 5' libraries via Polymerase Chain Reaction amplification using a Chromium Single-Cell V(D)J Enrichment kit (10x Genomics; Human T Cell:1000005, Human B Cell:1000006). The TCR/BCR sequences of each T/B cell were processed and clustered using the Cell Ranger VDJ pipeline (version 7.1.0), which enabled the identification of the CDR3 sequence and the rearranged TCR/BCR genes. Downstream analysis was performed using scRepertoire (version 1.10.1) (Borcherding et al., 2020) (https://github.com/ncborcherding/scRepertoire). The obtained barcoding information containing clonotype frequencies and indicators of TCR/BCR diversity was used to project T/B cells with dominant TCR/BCR clonotypes onto the UMAP plot (Fig. 1B

and Table S3).

Differential gene expression (DEG) analysis and functional enrichment

The *FindMarkers* function in Seurat was used to perform differential gene expression analysis (Finak et al., 2015). For each cluster, DEGs were identified by comparing two different groups. Only genes with adjust *P* value < 0.05 and an absolute value of $\log_2$ fold changes $|\log_2 FC| > 0.25$ were considered significant (test.use = "wilcox"). Functional enrichment of DEGs, which utilized gene sets from the Gene Ontology (GO) and the Kyoto Encyclopedia of Genes and Genomes (KEGG), was determined with Fisher's exact test as implemented in the clusterProfiler (version 4.8.3) (Wu et al., 2021) Bioconductor package.

Gene set enrichment analysis

Gene set enrichment analysis (GSEA) (version 1.2) (Subramanian et al., 2005) was performed to investigate pathway activity across different cell types. For each cell type, a pre-ranked gene list was created by sorting all genes by their $\log_2$FC values and this list was used as input for the GSEA package in pre-ranked mode. The annotation information of gene sets was downloaded from the Molecular Signatures Database (MSigDB) (https://www.gsea-msigdb.org/gsea/msigdb/index.jsp).

Calculation of signature scores

Signature scores for specific gene sets were calculated using the AUCell (version 1.22.0) (Aibar et al., 2017). First, a ranked expression matrix was built using the *AUCell_buildRankings* function. Then, the area under the curve (AUC) values were calculated using the *AUCell_calcAUC* function.

Transcription factor-target gene network analysis

Core regulatory TFs were predicted based on scRNA-seq data. TF binding motifs and their potential target genes were identified by the GENIE3 R/Bioconductor packages (version 1.22.0) (Huynh-Thu et al., 2010) and the RcisTarget database (version 1.20.0) (Verfaillie et al., 2015) of the SCENIC (version 1.2.4) (Aibar et al., 2017) workflow with default parameters (http://scenic.aertslab.org/). Only the TF-target with high-confidence annotations were selected for visualization with Cytoscape (version 3.9.1) (Shannon et al., 2003).

Cell fate trajectory analysis

Cell fate trajectory analysis was performed using the monocle2 (version 2.28.0) (Trapnell et al., 2014). First, DEGs across clusters were extracted using the *FindMarkers* function from Seurat. Then, cell ordering was performed based on these DEGs in an unsupervised fashion. Subsequently, the developmental trajectory was constructed after dimensionality reduction and cell ordering, using default parameters. Finally, putative trajectories were visualized using the *plot_cell_trajectory* function.

Ligand-receptor interaction analysis

Intercellular communication networks were inferred and analyzed using the CellChat (version 1.6.0) (Jin et al., 2025). The "Secreted Signaling" database was selected, and the pre-compiled human protein-protein interaction network was used as a priori information.

Disease-gene association and drug-gene interaction analysis

To test whether the SMEP is associated with vascular and immune system diseases, we utilized DisGeNET (version 7.3.0) (Piñero et al., 2021) via Cytoscape. We obtained genes associated with both vascular and immune system disease phenotypes, combined them into a gene signature (labeled as Shared_VID) and for subsequent GO enrichment analysis. To identify druggable genes in $CD14^{+}CD16^{+}$ Monocytes, we utilized the Drug-Gene Interaction database (DGIdb, version 4.2.0) (Freshour et al., 2021) which integrates information on the druggable genome from multiple drug databases. We filtered the drug-gene interactions to include only FDA-approved drugs that can be readily translated to the clinic.

Cytokine quantification

Cytokine concentrations in serum samples were quantified using the Cytometric Bead Array (CBA) method by CBA Flex Set (BD Biosciences, USA). Briefly, serum samples were centrifuged at 10,000 × g for 10 min to obtain clarified supernatants. For the assay, 50 µL of each sample (or standard), 50 µL of mixed capture bead suspension, and 50 µL of PE-conjugated detection antibody cocktail were co-incubated for 2 h at room temperature protected from light. Following incubation, the beads were washed twice via centrifugation (200 × g, 5 min) with wash buffer and finally resuspended in 300 µL of wash/assay buffer. Samples were analyzed with a flow cytometer. Bead populations corresponding to specific cytokines were discriminated based on their unique fluorescence signature. The median fluorescence intensity (MFI) in the PE channel was recorded. Cytokine concentrations in the samples were determined by interpolating the sample MFI values against a standard curve generated from the cytokine standards.

CFSE-based antigen presentation assay

Antigen presentation capacity was assessed by measuring the ability of monocytes to stimulate $CD4^{+}$ T-cell proliferation. Briefly, $CD14^{+}$ monocytes were isolated from peripheral whole blood of LMS patients using $CD14^{+}$ MicroBeads (Miltenyi Biotec, Germany) according to the manufacturer's instructions, with monocytes isolated from healthy children serving as controls. $CD4^{+}$ T cells were isolated from another healthy donors using $CD4^{+}$ MicroBeads (Miltenyi Biotec, Germany), labeled with carboxyfluorescein succinimidyl ester (CFSE, BD Biosciences, USA), and co-cultured with the isolated monocytes (from either LMs patients or controls) for 3 days. Anti-CD3 and anti-CD28 antibodies (BD Biosciences, USA) were used as stimulators. $CD4^{+}$ T-cells proliferation was assessed by measuring CFSE dilution using a BD FACSCanto™ II flow cytometer and analyzed using BD FACSDiva™ software

(version 3.1; BD Biosciences, USA).

Imaging flow cytometry

The interaction of monocyte-derived CXCL16 with CXCR6 on MAIT and T cells was analyzed by imaging flow cytometry. Briefly, peripheral blood was incubated with a pre-mixed fluorescently labeled antibody cocktail for 20 minutes in the dark. Cells were washed twice with PBS and resuspended in buffer for acquisition. Acquisition was performed with ImageStream X Mk II (Amnis) and INSPIRE software at 40X magnification. Data analysis was performed using IDEAS (version 6.2.64). Gating strategies: Monocytes: $CD45^{+}CD14^{+}$, T cells: $CD45^{+}CD3^{+}$, MAIT cells: $CD45^{+}CD3^{+}CD161^{hi}TCRV\alpha7.2^{+}$. All antibodies were purchased from BD Biosciences. Experiments followed applicable guidelines and regulations.

Whole-mount immunostaining

Bilateral auricular skin sheets were dissected from cartilage in physiological saline under a stereo microscope. Tissues were fixed in 4% PFA for 2 hours at room temperature, washed with PBS (3×5 min) on an orbital shaker, and permeabilized/blocked in PBS containing 1% BSA and 1% Triton X-100 for 4 hours at room temperature with shaking. Samples were incubated with rabbit anti-mouse LYVE-1 antibody (Abcam; 1:150 in PBS/2% BSA) at 4°C for 40 hours without agitation. Following five 5-min PBS washes with shaking, tissues were incubated with Alexa Fluor 488-conjugated goat anti-rabbit IgG (Invitrogen; 1:500 in PBS/2% BSA) at 4°C for 12 hours without agitation, followed by five 10-min PBS washes with shaking. Tissues were flat-mounted on glass slides using 50% glycerol, coverslipped, and imaged via fluorescence microscopy.

Generate heterozygous Vegfr3 knockout mice

Male Vegfr3 $flox^{+/-}$ heterozygous mice, female CMV-cre mice were obtained from the Nanjing Institute of Biomedicine. Vegfr3 $flox^{+/-}$ heterozygous mice were mated with CMV-cre mice to generate $Vegfr3^{+/-}CMV\text{-}cre^{+}$ mice. After reaching sexual maturity, these $Vegfr3^{+/-}CMV\text{-}cre^{+}$ mice were brother and sister mated. $Vegfr3^{+/-}CMV\text{-}cre^{+}$ (abbreviated as $Vegfr3^{+/-}$) mice were obtained. No $Vegfr3^{-/-}$ newborns were found from the crosses of these surviving $Vegfr3^{+/-}$ animals, indicating that the absence of VEGFR3 is embryonic lethal. All mouse breeding and experimental procedures were conducted in the Specific Pathogen-Free (SPF) Laboratory Animal Center at the Capital Institute of Pediatrics. Animal handling complied strictly adhered to the NIH Guidelines for the Animal Care and Use and the institutional animal welfare regulations of the Capital Institute of Pediatrics (Ethical Approval Code: DWLL2019003).

Treatment of S100A8 inhibitor paquinimod

Paquinimod is an inhibitor of S100A8 (Tahvili et al., 2018). For paquinimod treatment, the compound (Med- Chem Express, HY-100442) was sequentially dissolved in 2% DMSO, 30% PEG300, 5% Tween80, and ddH_2O mice were administrated at the concentration of 1 mg/kg/day by oral gavage three times a week as indicated. The control group was given an equal volume of normal saline. Under normal diet (ND) feeding, 2-month-old male mice were treated with paquinimod or vehicle for 4 weeks.

Statistical analysis

For the analysis of cell percentages, cytokines concentrations, gene set scores and $CD4^+$ T-cell proliferation, statistical analysis was performed two-sided Wilcoxon rank-sum tests. For lymphatic vessel diameter, statistical analysis was performed unpaired t-test. A P value < 0.05 was considered statistically significant. *, **, *** and **** indicate $P < 0.05$, $P < 0.01$, $P < 0.001$ and $P < 0.0001$, respectively; "ns" indicates not significant.

Reference

Aibar S, Gonzalez-Blas C.B, Moerman T et al. SCENIC: single-cell regulatory network inference and clustering. *Nat Methods* 2017; **14**: 1083-1086.

Borcherding N, Bormann N.L, and Kraus G. scRepertoire: An R-based toolkit for single-cell immune receptor analysis. *F1000Res* 2020; **9**: 47.

DePristo M.A, Banks E, Poplin R et al. A framework for variation discovery and genotyping using next-generation DNA sequencing data. *Nat Genet* 2011; **43**: 491-498.

Finak G, McDavid A, Yajima M et al. MAST: a flexible statistical framework for assessing transcriptional changes and characterizing heterogeneity in single-cell RNA sequencing data. *Genome Biol* 2015; **16**: 278.

Freshour S.L, Kiwala S, Cotto K.C, et al. Integration of the Drug-Gene Interaction Database (DGIdb 4.0) with open crowdsource efforts. *Nucleic Acids Res* 2021; **49**: D1144-d1151.

Hao Y, Hao S, Andersen-Nissen E et al. Integrated analysis of multimodal single-cell data. *Cell* 2021; **184**: 3573-3587.e3529.

Huynh-Thu V.A, Irrthum A, Wehenkel L et al. Inferring regulatory networks from expression data using tree-based methods. *PLoS One* 2010; **5**: e12776.

Jin S, Plikus M.V, and Nie Q. CellChat for systematic analysis of cell-cell communication from single-cell transcriptomics. *Nat Protoc* 2025; **20**: 180-219.

Piñero J, Saüch J, Sanz F et al. The DisGeNET cytoscape app: Exploring and visualizing disease genomics data. *Comput Struct Biotechnol J* 2021; **19**: 2960-2967.

Shannon P, Markiel A, Ozier O et al. Cytoscape: a software environment for integrated models of biomolecular interaction networks. *Genome Res* 2003; **13**: 2498-2504.

Stuart T, Butler A, Hoffman P et al. Comprehensive Integration of Single-Cell Data. *Cell* 2019; **177**: 1888-1902.e1821.

Subramanian A, Tamayo P, Mootha V.K et al. Gene set enrichment analysis: a

knowledge-based approach for interpreting genome-wide expression profiles. *Proc Natl Acad Sci U S A* 2005; **102**: 15545-15550.

Tahvili S, Törngren M, Holmberg D et al. Paquinimod prevents development of diabetes in the non-obese diabetic (NOD) mouse. *PLoS One* 2018; **13**: e0196598.

Trapnell C, Cacchiarelli D, Grimsby J et al. The dynamics and regulators of cell fate decisions are revealed by pseudotemporal ordering of single cells. *Nat Biotechnol* 2014; **32**: 381-386.

Verfaillie A, Imrichova H, Janky R et al. iRegulon and i-cisTarget: Reconstructing Regulatory Networks Using Motif and Track Enrichment. *Curr Protoc Bioinformatics* 2015; **52**: 2.16.11-12.16.39.

Wang K, Li M, and Hakonarson H. ANNOVAR: functional annotation of genetic variants from high-throughput sequencing data. *Nucleic Acids Res* 2010; **38**: e164.

Wu T, Hu E, Xu S et al. clusterProfiler 4.0: A universal enrichment tool for interpreting omics data. *Innovation (Camb)* 2021; **2**: 100141.

Zheng L, Qin S, Si W et al. Pan-cancer single-cell landscape of tumor-infiltrating T cells. *Science* 2021; **374**: abe6474.

Figure S1

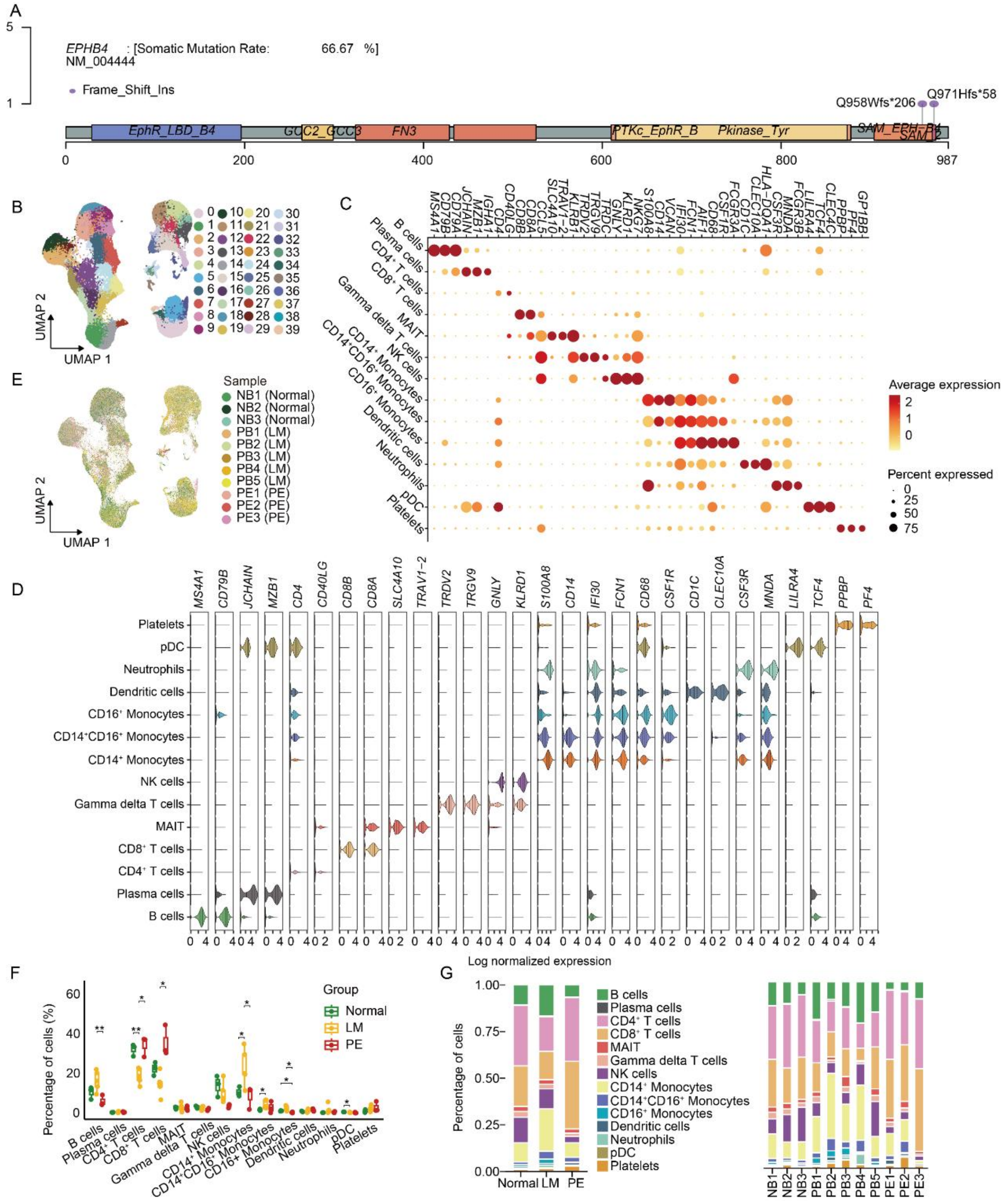

Figure S1. Single-cell transcriptomic analysis of immune cells from peripheral blood and pleural effusion samples. (A) The lollipop plot illustrating the location and type of somatic mutations identified in the *EPHB4* gene in patients with LMs. (B) UMAP plot showing the distribution of cells across different clusters. (C and D) Dot plot (C) and violin plot (D) showing the expression levels of marker genes of each cell type. (E) UMAP plot showing the distribution of cells across different samples. (F) Box plots showing the percentage of each cell type across the three groups, (Normal: $n = 3$, LM: $n = 5$, PE: $n = 3$). Statistical significance was determined using an unpaired two-sided t-test. (G) Stacked bar plots showing the composition of cell types across groups (left) and samples (right). *$P < 0.05$, **$P < 0.01$.

Figure S2

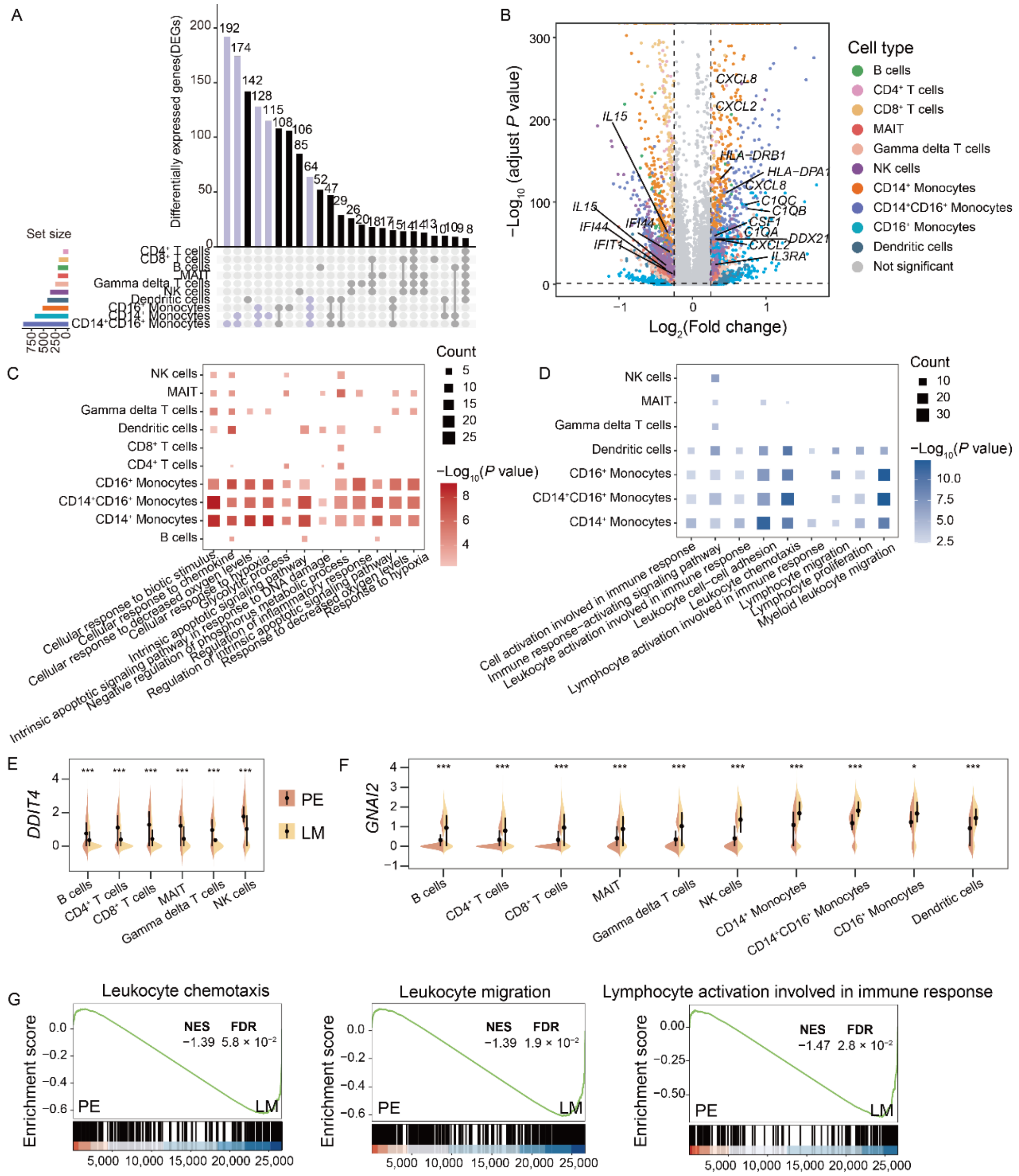

Figure S2. Transcriptional alterations in immune functions across major cell types between the LM and PE groups. (A) UpSet plot showing the intersections of DEGs among major immune cell types between the LM and PE groups. Dots indicate shared genes among cell types. The upper bars show intersection sizes, the left bars show total gene counts per cell type, and representative key genes are highlighted above. (B) Volcano plot displaying DEGs across cell types. Each point represents an individual gene, colored by its associated cell type. Gray points denote genes without significant expression differences between the LM and PE groups. (C and D) Heatmap showing significantly enriched GO terms representing disease-related pathways that were upregulated (C) or downregulated (D) in the PE group compared with the LM group. (E and F) Differential expression of *DDIT4* (E) or *GNAI2* (F) between the PE and LM groups in each cell type, two-sided Wilcoxon rank-sum tests. (G) GSEA plots showing

pathway enrichment scores in the PE group compared with the LM group. *$P < 0.05$, **$P < 0.01$, ***$P < 0.001$.

Figure S3

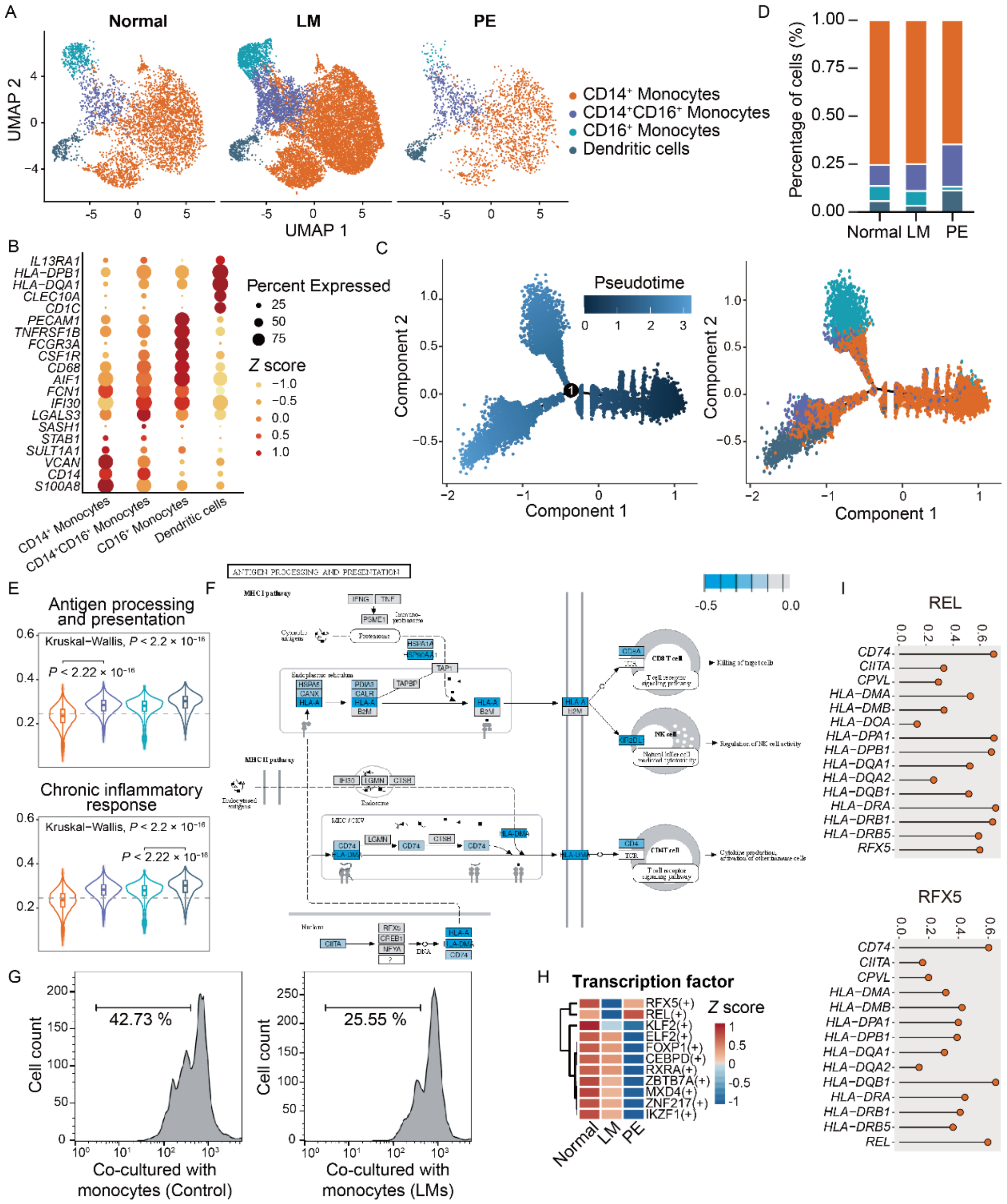

Figure S3. Characterization of myeloid cells in the LMs. (A) UMAP plots showing the distribution of myeloid cell subtypes across the three groups. (B) Dotplot showing the expression levels of marker genes specific to each myeloid cell subtype. (C) Trajectory inference of myeloid cells, colored by pseudotime (left) and subtype (right). (D) Stacked bar plot showing the proportion of each myeloid cell subtype across each group. (E) Violin plots showing pathway enrichment scores across myeloid cell subtypes. Statistical significance was assessed using Kruskal-Wallis tests for overall group differences, with post-hoc Wilcoxon rank-sum tests for pairwise comparisons between designated subtypes. (F) KEGG pathway diagram showing downregulated DEGs included in the "Antigen processing and presentation" pathway. (G) Representative CFSE histograms of gated CD3⁺CD4⁺ T cells from one donor in each

group (Control and LMs) showing fluorescence intensity. (H) Heatmap showing the activity of differentially expressed TFs in the CD14$^+$ monocytes across the three groups. The color scale represents the *Z* score of TF activity. (I) Correlation between the regulator activity of TFs (REL and RFX5) and the expression of genes, which were involved in "Antigen processing and presentation" KEGG pathway. Spearman correlation coefficients and two-tailed *P* values.

Figure S4

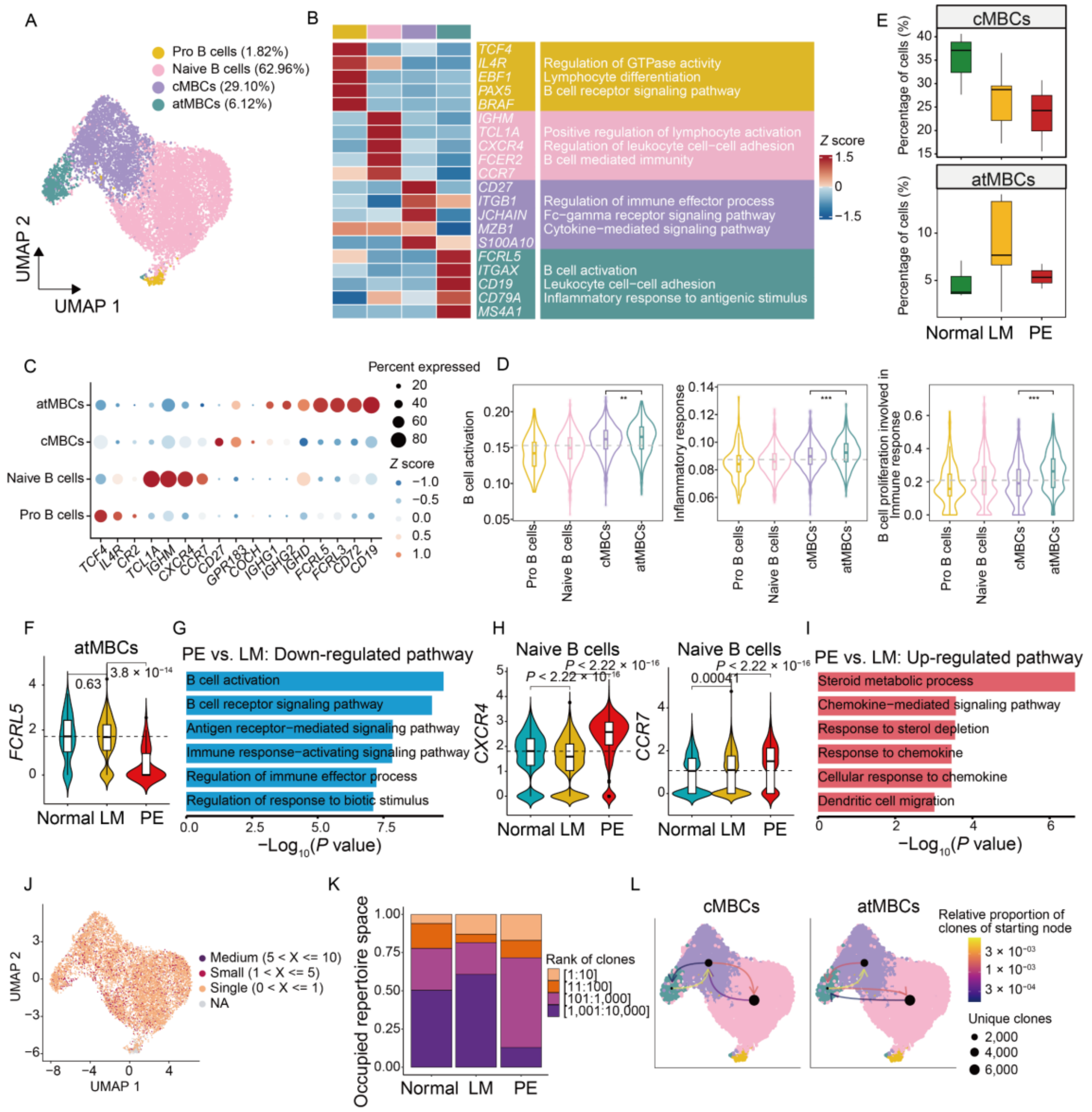

Figure S4. Dysfunctional atypical memory B cells abnormally expand in LMs. (A) UMAP plot showing four subtypes of B cells identified by unsupervised re-clustering. (B) Heatmap showing relative expression levels of canonical marker genes and representative functional signatures (derived from GO enrichment analysis of hypervariable genes) across B cell subtypes. (C) Dot plot showing the expression patterns of immunoglobulin genes and functional genes across each B cell subtype. (D) Violin plots showing pathway scores across the three groups, two-sided Wilcoxon rank-sum tests. (E) Box plots showing the percentage of two B cell subtypes across the three groups. (F) Violin plots showing the expression level of *FCRL5* in atMBCs across the three groups, two-sided Wilcoxon rank-sum tests. (G) Bar plot demonstrating the downregulated functional pathways of atMBCs in the PE group compared with the LM group. (H) Violin plots showing the expression levels of *CXCR4* (left) and *CCR7* (right) in naïve B cells across the three groups, two-sided Wilcoxon rank-sum tests. (I) Bar plot showing the upregulated functional pathways in naïve B cells in the PE group compared with the LM group. (J) UMAP plot showing B cell subtypes integrated with BCR clonality. Color indicates groups based on the frequency of clonotypes in total

cells. (K) Stacked bar plot showing the percentage distribution of BCR clone ranks across the three groups. (L) Density plots showing the relative proportion of clones at the starting node in cMBCs (left) and atMBCs (right). $*P < 0.05$, $**P < 0.01$, $***P < 0.001$.

Figure S5

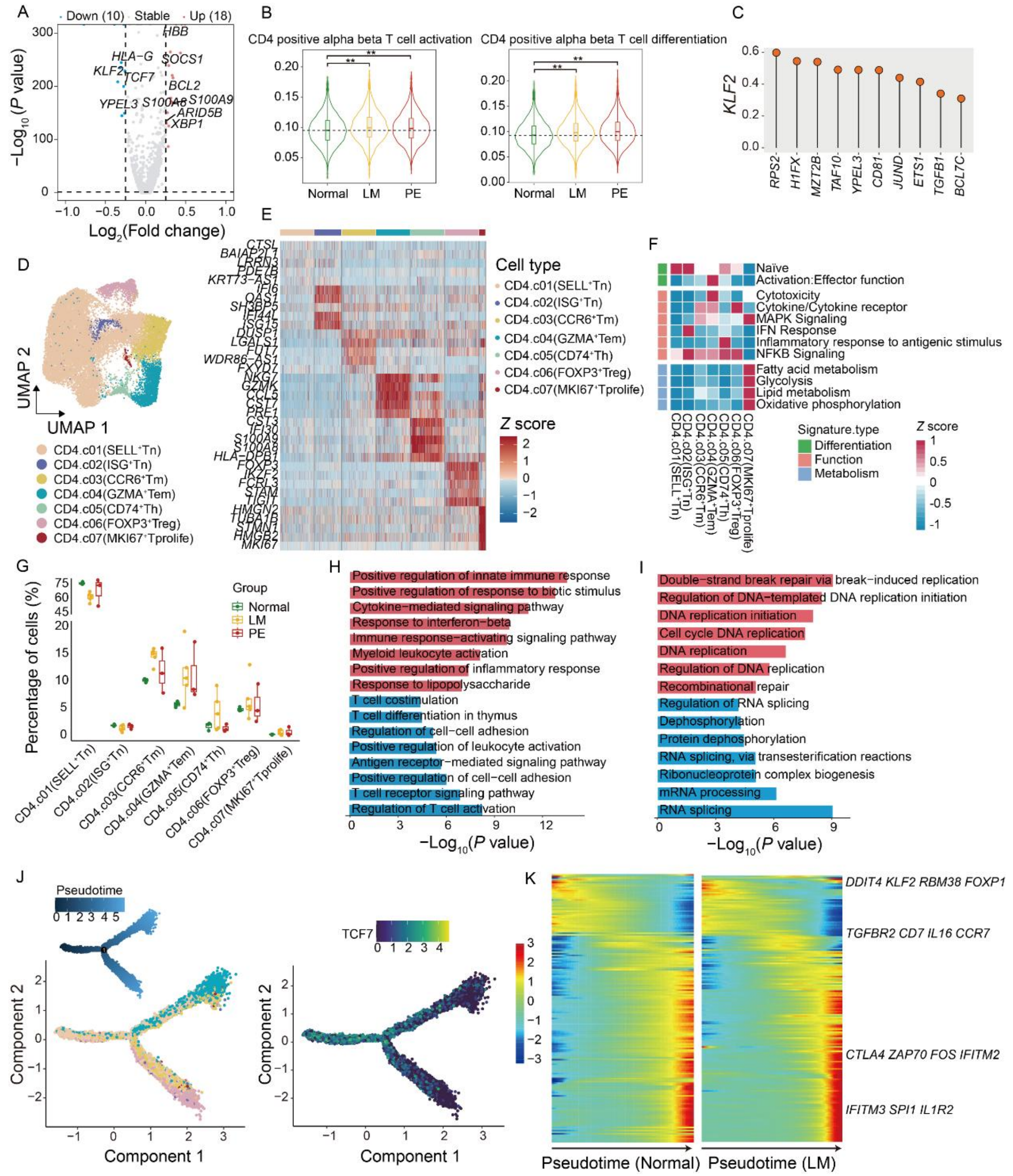

Figure S5. Transcriptional landscape of CD4⁺ T cells in the immune microenvironment of LMs. (A) Volcano plot showing DEGs of CD4⁺ T cells compared between the Normal and the LM group. Genes with an adjusted *P* value < 0.05 and $|\log_2 FC| > 0.25$ are considered significant. (B) Violin plots showing the gene set scores across the three groups, two-sided unpaired Wilcoxon test. (C) Lollipop plot showing genes that exhibit a significant positive correlation with the expression of *KLF2*, with Spearman's correlation coefficients and two-tailed *P* values. (D) UMAP plot showing seven CD4⁺ T-cell subtypes by re-clustering. (E) Heatmap showing the expression of top five marker genes in each subtype. (F) Heatmap illustrating 12 curated pathway scores (gene sets from the study with PMID: 37248301) across CD4⁺ T-cell subtypes. (G) The box plots showing the proportion of CD4⁺ T-cell subtypes across the three groups. (H and I) The bar plots showing GO terms of disease-related upregulated

(red) and downregulated (blue) pathways between the Normal and LM groups in CD4.c05(CD74^{+} Th) (H) and CD4.c07(MKI67^{+} T prolife) (I). (J) Dim plots (left) showing the pseudotime of the CD4^{+} T lineage cells. The points are colored by pseudotime (top) and cell types (bottom). Dim plot (right) showing the expression of *TCF7* along the trajectory of the CD4^{+} T lineage cells. (K) Heatmap depicting the expression patterns of 190 genes that exhibit significantly different expression patterns along the cell differentiation trajectory between two conditions. The significance threshold was set as an adjusted P value < 0.05. *$P < 0.05$, **$P < 0.01$.

Figure S6

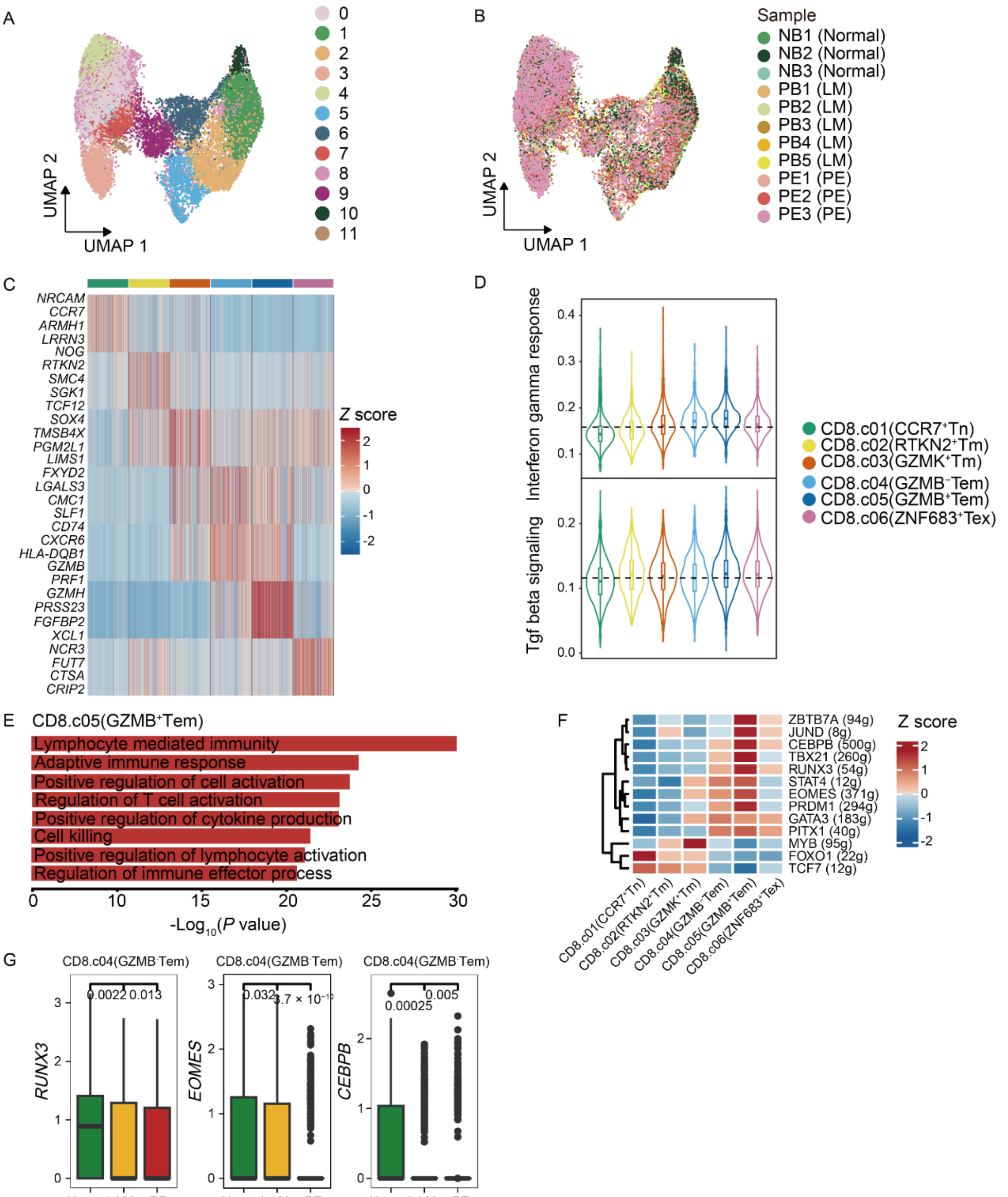

Figure S6. Cell-type specific transcriptional profiling in CD8+ T cells. (A) UMAP plot showing the distribution of CD8+ T-cell clusters by re-clustering. (B) UMAP plot showing cell distribution among each sample. (C) Heatmap showing the expression levels of canonical marker genes used to define subtypes. (D) Violin and box plots showing the functional scores across CD8+ T-cell subtypes. (E) Bar plot demonstrating the upregulated functions in GZMB+CD8+ T cells. (F) Heatmap showing the activity of TFs across CD8+ T-cell subtypes. (G) Boxplots showing the expression of *RUNX3*, *EOMES* and *CEBPB* in GZMB-CD8-T cells across the three groups.

Figure S7

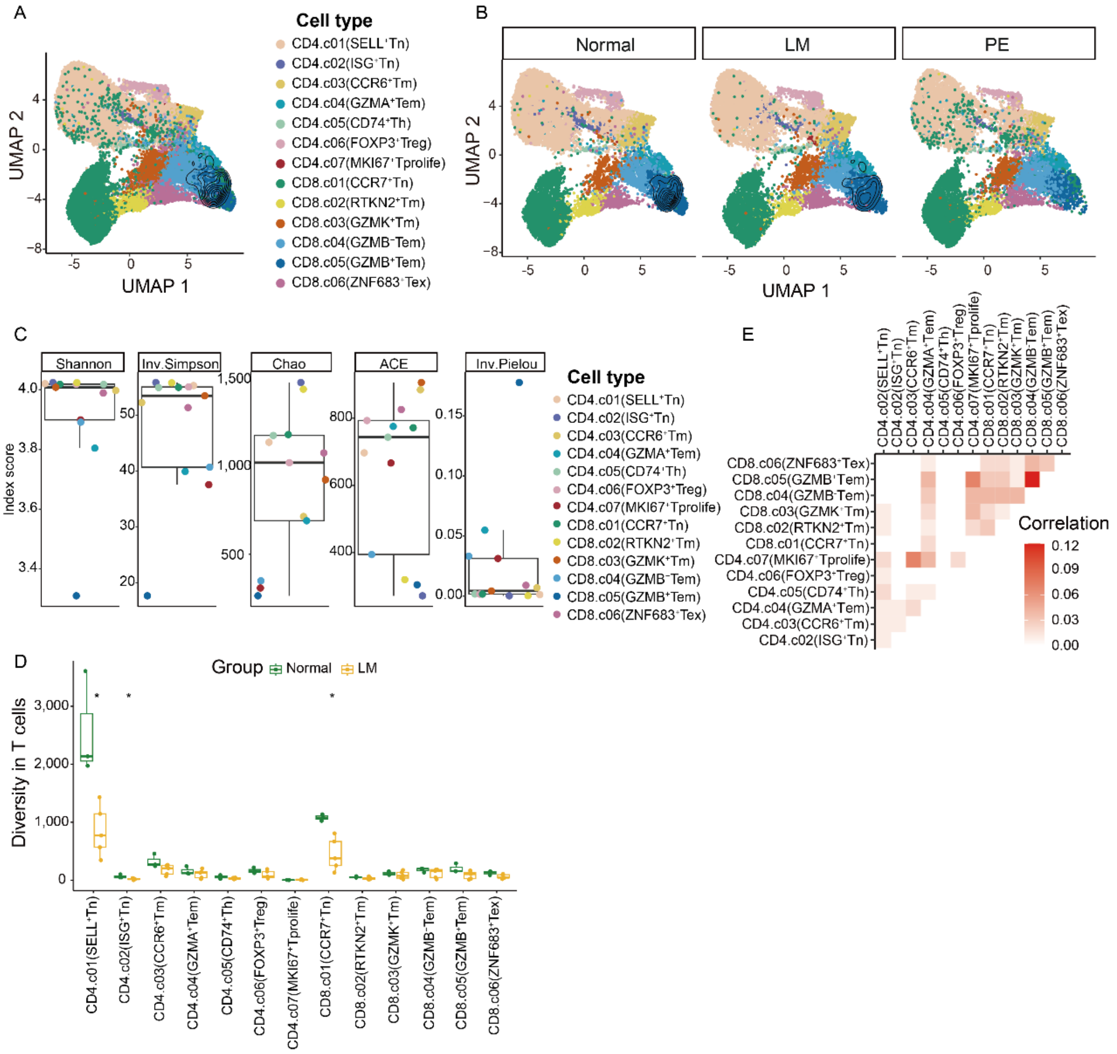

Figure S7. T-cell receptor clonality and diversity analysis. (A) UMAP plot showing integrated T-cell subtypes. The circled region in the plot highlights a subtype of highly clonally expanded T cells. (B) UMAP plots showing integrated T-cell subtypes among three groups respectively. The circled region in the plot highlights a subtype of highly clonally expanded T cells. (C) TCR repertoire diversity estimation of T-cell subtypes. The Shannon index is a metric for quantifying clonotype diversity of the detected TCRs-higher index means higher diversity. Inv.Simpson is a metric quantifying TCR clonotype diversity. Number of clonotypes estimated by Chao indicates the predicated theoretical clonotype diversity. A higher index indicates greater diversity, reflecting a more even distribution of clonotypes in the repertoire. ACE (Abundance-based Coverage Estimator) estimates the total number of clonotypes based on their abundance. In TCR analysis, a higher ACE value suggests greater predicted theoretical clonotype diversity, accounting for both observed and potentially undetected clonotypes. Inv.Pielou assesses the evenness of TCR clonotype distribution. (D) Box plot showing diversity of TCR in T-cell subtypes among the three groups. *t* test (unpaired, two-sided; Normal: $n = 3$, LM: $n = 5$, PE: $n = 3$). (E) Heatmap showing the TCR clone similarity among T-cell subtypes. *$P < 0.05$.

Figure S8

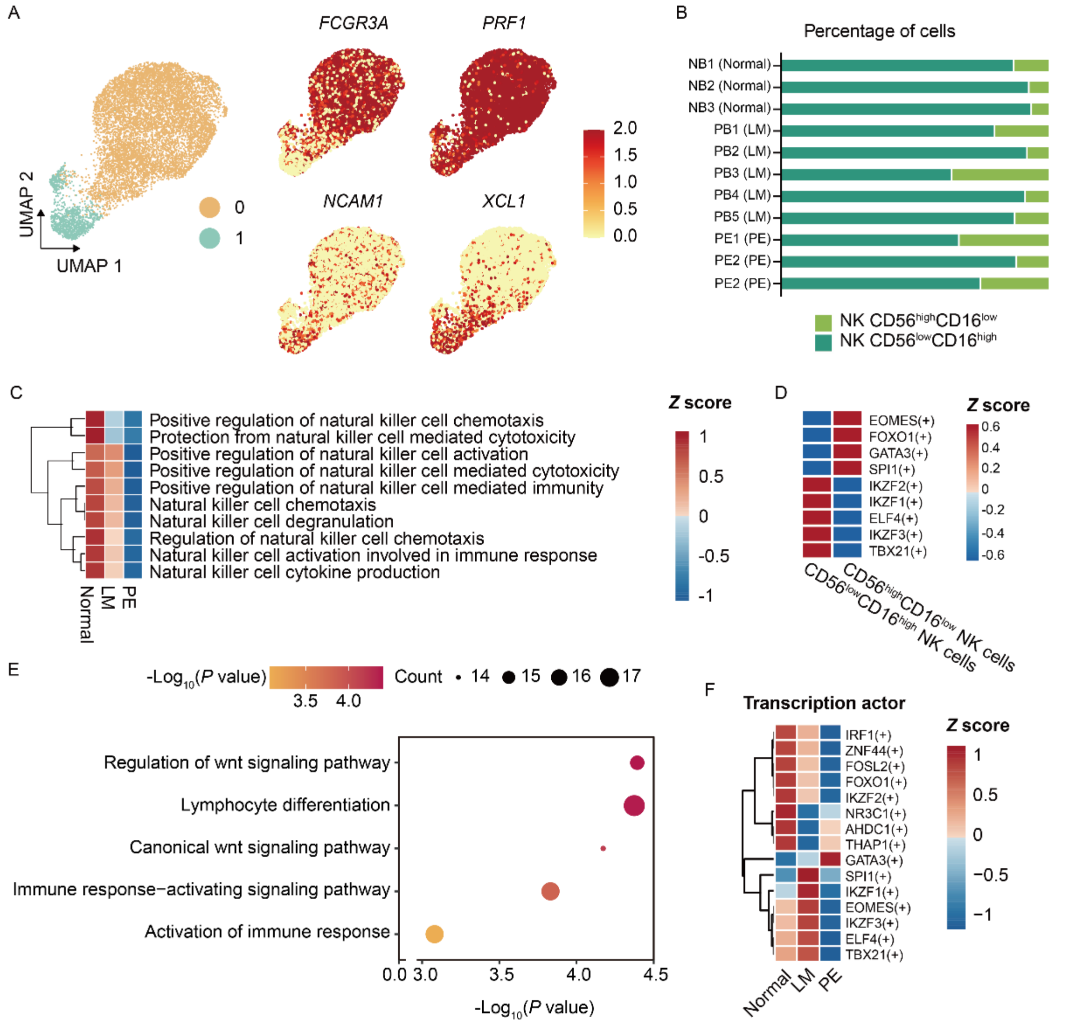

Figure S8. Profiling the NK cell in LMs at single-cell level. (A) UMAP plot (left) showing the distribution of NK cell clusters by re-clustering. UMAP plots (right) showing the expression levels of marker genes. (B) Stacked bar plot showing the percentage of subtypes across each sample. (C) Heatmap showing pathway scores in all NK cells across the three groups. (D) Heatmap showing the activity of TFs in two subtypes. (E) KEGG pathways enriched in target genes that regulated by selected TFs in Fig. 6F, $P < 0.05$. (F) Heatmap showing the activity of selected TFs in each group.

Figure S9

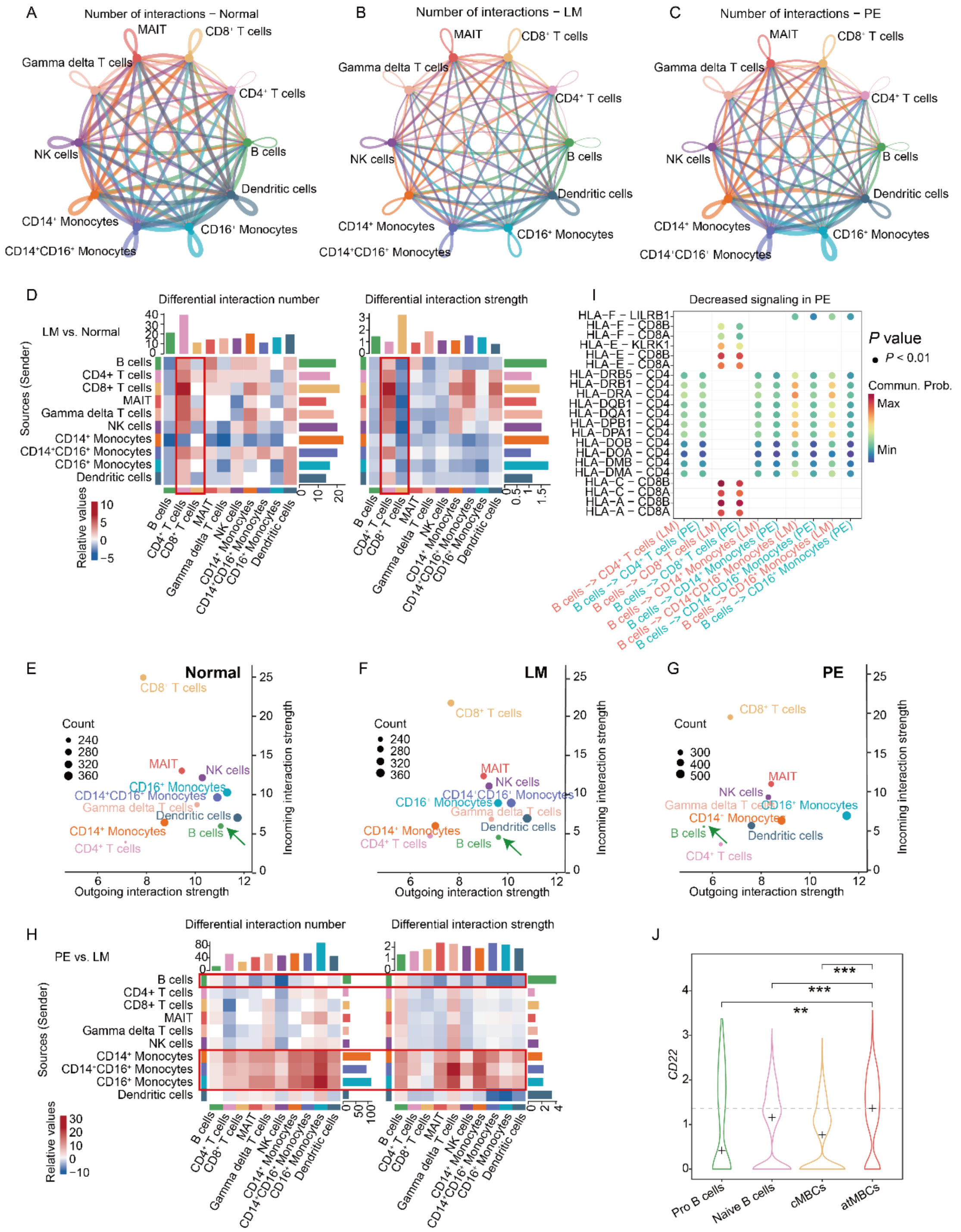

Figure S9. Dynamics of intercellular interaction networks in the immune microenvironment. (A-C) Circle plots showing the communication frequency among different cell types in the Normal (A), LM (B) and PE (C) groups. (D) Heatmaps showing the differential interaction number (left) and strength (right) between the LM and Normal groups. (E - G) Cell-cell interaction strengths for all cell types indicating incoming and outgoing interactions in the Normal (E), LM (F) and PE (G) groups. (H) Heatmaps showing the differential interaction number (left) and strength (right) between PE and LM groups. (I) Comparison of the significant LR pairs between the PE

and LM groups, which contribute to the signaling to B cells. Dot color reflects communication probabilities and dot size represents computed *P values*. Empty space means the communication probability is zero. *P values* are computed from one-sided permutation test. (J) Violin plot showing the *CD22* gene expression among B cell subtypes. "+" represents mean expression value of each subtype and the dashed line represents the mean expression value of atMBCs. Two-sided unpaired Wilcoxon test. $*P < 0.05$, $**P < 0.01$, $***P < 0.001$.

Figure S10

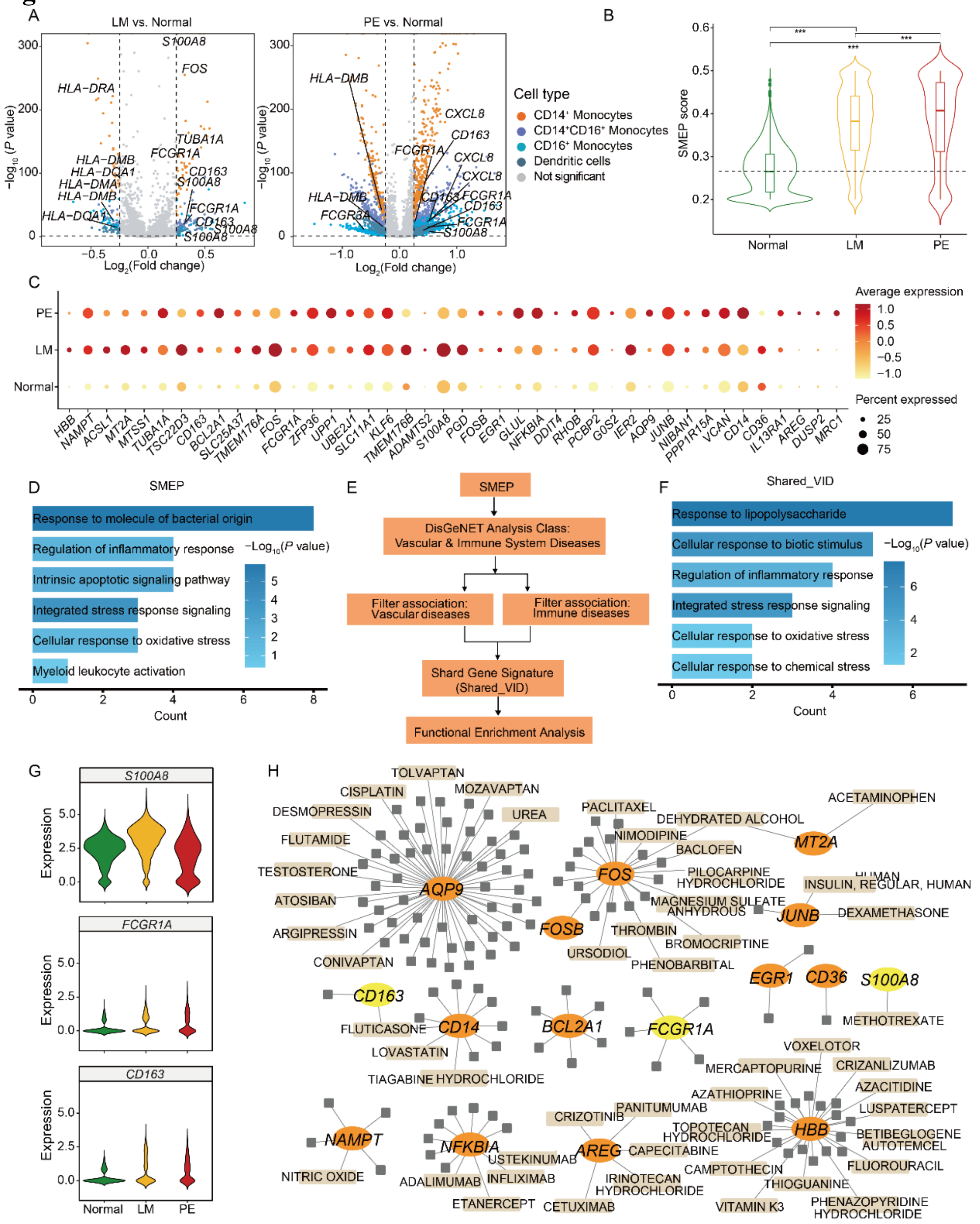

Figure S10. Association of SMEP with vascular and immune system diseases. (A) Volcano plots showing the DEGs between the Normal group versus the LM group (left) and PE group (right), respectively. (B) Violin plot showing the SMEP score across the three groups. Two-sided unpaired Wilcoxon test. (C) Dot plot showing the gene expression signatures of SMEP across the three groups. (D) Bar plot demonstrating the GO enrichment pathways of SMEP, $P < 0.05$. (E) Overview of the workflow for the identification of target genes associated with both vascular and immune diseases. (F) Bar plot demonstrating the GO enrichment pathways of Shared-VID, $P < 0.05$. (G) Violin plots showing the expression of *S100A8*, *FCGR1A* and *CD163* in $CD14^{+}CD16^{+}$ Monocytes across the three groups. (H) Drug-gene interaction network visualization of

all the druggable targets in SMEP. The nodes in circle are gene targets, whereas the nodes in square are FDA-approved drugs. *$P < 0.05$, **$P < 0.01$, ***$P < 0.001$.

Figure S11

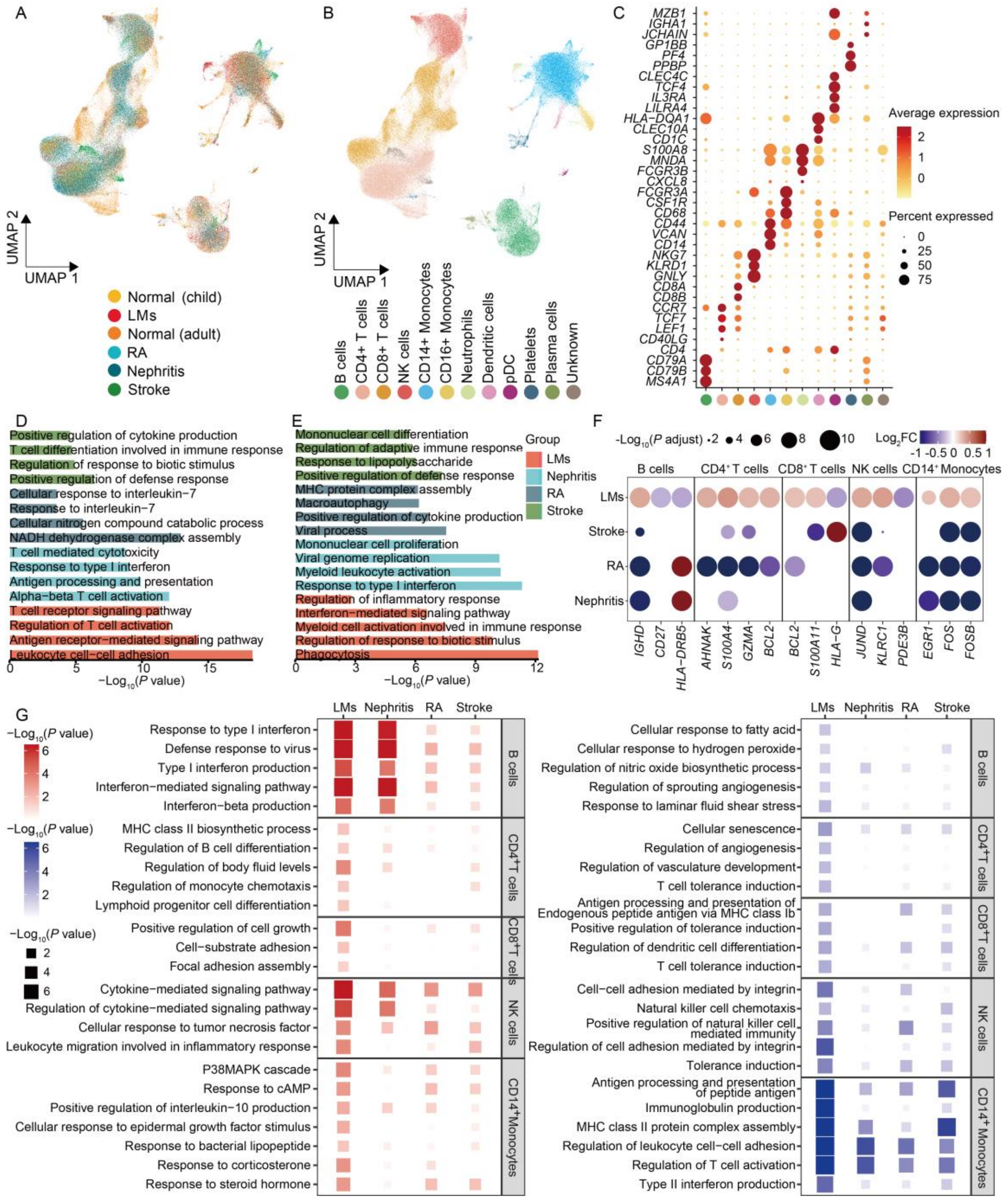

Figure S11. Immune cell phenotypes and dysregulated pathways of LMs among diverse immune-mediated diseases. (A) UMAP embedding of 188,974 cells from two normal groups and four disease groups. LMs： lymphatic malformations. RA: rheumatoid arthritis. (B) UMAP embedding of 11 cell types and unassigned type. pDC: plasmacytoid dendritic cell. (C) Dot plot showing top marker genes for each cell type. (D and E) GO functional enrichment of high-expression genes identified across four diseases in T cells (D) or monocytes (E). (F) Dot plot showing level (fold-change; Log_2FC) and significance (-Log_{10} (adjust *P* value)) of selected DEGs in five cell types across the four diseases. (G) Differentially activated pathways ($P < 0.05$) enriched in upregulated (left) or downregulated DEGs (right) across the four diseases.
